# Characteristics of peripheral blood neutrophil subsets in patients with primary Sjogren’s syndrome based on single-cell RNA sequencing

**DOI:** 10.3389/fimmu.2026.1820004

**Published:** 2026-05-04

**Authors:** Zhaomeng Gao, Jian Zheng, Xueling Ma, Yuanwei Han, Jia Wang, Li Fang, Hong Zhu, Xiaoyu Zhang

**Affiliations:** 1 The First Clinical Medical College, Ningxia Medical University, Yinchuan, China; 2 Department of Rheumatology, Zibo Central Hospital, Zibo, China; 3 Biochip Center, General Hospital of Ningxia Medical University, Yinchuan, China; 4 School of Health Management, Ningxia Vocational and Technical College for Minorities, Wuzhong, China; 5 School of Nursing, Ningxia Medical University, Yinchuan, China; 6 Department of Rheumatology, General Hospital of Ningxia Medical University, Yinchuan, China; 7 Department of Rheumatology, Affiliated Hospital of Qingdao University, Qingdao, Shandong, China

**Keywords:** CLEC12A, LY6E, neutrophil, primary, single-cell RNA sequencing, Sjogren’s syndrome

## Abstract

**Background:**

Neutrophils, which are polymorphonuclear leukocytes of the phagocytic system, serve as critical mediators of inflammation-induced injury and are implicated in various diseases. However, the role of neutrophils in primary Sjögren’s syndrome (pSS) remains underexplored.

**Objectives:**

Single-cell RNA sequencing (scRNA-seq) was used to construct a map of neutrophil subsets and cell interactions in pSS, and to explore the roles and mechanisms of each subset in the development and progression of pSS, thereby providing new theoretical support for the pathogenesis of pSS and clinical drug development.

**Methods:**

Include newly diagnosed patients with pSS who presented to the Rheumatology and Immunology Department of Ningxia Medical University General Hospital between December 2022 and January 2025. Peripheral blood neutrophils were obtained from pSS and healthy control (HC) subjects. Following quality control, dimensionality reduction, clustering, and cell annotation, we analyzed compositional and distributional differences in neutrophil subsets between the pSS and HC groups. Significantly differentially expressed genes (DEGs) in neutrophils underwent Gene Ontology (GO) enrichment analysis and Kyoto Encyclopedia of Genes and Genomes (KEGG) pathway enrichment analysis, along with molecular interaction and pseudo-time analysis between cells. The DEGs *LY6E* and *CLEC12A* were further verified by RT-qPCR. Statistical analysis was performed using the non-paired two-tailed Wilcoxon rank-sum test, Mann-Whitney U test, and non-paired two-tailed Student’s t-test. Visualization was conducted using Monocle and CellphoneDB software.

**Results:**

Neutrophil counts were significantly lower in the pSS group compared to the HC group. Neutrophils were further clustered into six subsets (Neutrophil 1, Neutrophil 2, Neutrophil 3, Neutrophil 4, Neutrophil 5, and Neutrophil 6), with Neutrophil 2 exhibiting distinct characteristics. The DEGs in neutrophils were primarily involved in biological processes such as viral response and defense, type I interferon signaling pathways, and were enriched in signaling pathways including interferon-α response and inflammatory response. Neutrophils interact with natural killer (NK) cells and plasmacytoid dendritic cells (pDCs) via CCL5-CCR1, LGALS9-HAVCR2, FTH1-SCARA5, and FTL-SCARA5. Pseudo-time analysis revealed that neutrophils first differentiate into the Neutrophil 2 subset and ultimately into the Neutrophil 4 subset. This progression is characterized by progressively enhanced cellular signaling and behavioral regulation capabilities, while concurrently diminishing immune defense responses and neutrophil-mediated immune functions.

**Conclusion:**

Each subset of neutrophils is involved in the occurrence and development of pSS disease at different stages. In particular, *LY6E* and *CLEC12A* among the DEGs specifically upregulated by pSS neutrophils may be biomarkers and potential therapeutic targets for pSS.

## 1 Introduction

Sjogren’s syndrome (SS) is a chronic systemic autoimmune disease characterized by progressive lymphocyte infiltration of exocrine glands (mainly salivary glands and lacrimal glands), which can cause a decrease in exocrine gland function, leading to xerophthalmia and xerostomia (1). The pathogenesis of primary Sjögren’s syndrome (pSS) involves the secretion of multiple cytokines produced by activated neutrophils, which together lead to tissue damage and functional impairment.

Single-cell RNA sequencing (scRNA-seq) is an emerging technology for studying the transcriptome profile of specific tissues. It can analyze transcriptome characteristics at the single-cell level and is suitable for exploring complex tissue microenvironments and cell heterogeneity (2). This technology has been used in the study of a variety of autoimmune diseases. It can not only characterize single cells but also assist in identifying new cell subsets, analyzing their biological significance, and elucidating cell differentiation trajectories and immunopathogenic mechanisms. It is a powerful means to analyze the pathogenesis of pSS.

A study by Peng et al. demonstrated that the type I interferon signaling pathway is activated in neutrophils from patients with pSS, and that type I interferon-related genes are highly expressed. *In vitro* studies have confirmed that the activation of type I interferon (IFN) in neutrophils from pSS patients can induce mitochondrial damage and the production of associated reactive oxygen species (ROS), ultimately leading to the formation of neutrophil extracellular traps (NETs) (3). NETs contain various self-antigens, such as DNA, histones, and myeloperoxidase, which can be recognized by the immune system and trigger an autoimmune response. Compared with the control group, patients with pSS have significantly elevated levels of NETosis markers in their plasma and labial glands, and these levels correlate with disease activity. In the salivary glands of patients with pSS, there is a significant increase in the expression of neutrophil-associated cytokines and their receptors. The interaction pattern between neutrophils and CD4+ T cells has shifted from an engagement pattern to an avoidance pattern; this shift suggests the heterogeneity of the pSS microenvironment (4). In addition, pSS patients are accompanied by severe neutropenia, which is characterized by a basically asymptomatic course and fluctuation. 14%-42% of pSS cases develop leukopenia (5). The mechanism of neutropenia in patients with pSS is unknown.

This study employed scRNA-seq to construct a neutrophil subset atlas in pSS, elucidating the roles and mechanisms of each neutrophil subset in pSS pathogenesis and progression. It further investigated interactions among these subsets and analyzed the expression of neutrophil-associated differentially expressed genes (DEGs) in pSS patients. These findings provide novel theoretical support for pSS pathogenesis research and clinical drug development.

## 2 Materials and methods

### 2.1 General information

This study included 34 patients with newly diagnosed pSS who were diagnosed between December 2022 and January 2025 at the Department of Rheumatology and Immunology of Ningxia Medical University General Hospital. All patients met the 2016 American College of Rheumatology (ACR) or European League Against Rheumatism (EULAR) classification criteria (6). These patients constituted the disease group (pSS group), with a mean age of 46.47 ± 11.00 years (range 24–67 years). The healthy control (HC) group consisted of 23 healthy individuals who underwent physical examinations at Ningxia Medical University General Hospital during the same period, with a mean age of 41.57 ± 11.04 years (range 26–60 years) and had no history of autoimmune diseases or other diseases. All the participants were female, and written informed consent was obtained. Peripheral venous blood samples and clinically related data were collected. Neither group of subjects received glucocorticoids (GCs) or immunosuppressive treatment before blood collection. Three pSS samples and three HC samples were randomly selected for scRNA-seq. All research procedures were conducted in accordance with the Declaration of Helsinki (2022 revision) and approved by the Ethics Committee of the General Hospital of Ningxia Medical University (Approval No.: KYLL-2024-0327).

#### 2.1.1 Inclusion criteria

① Meet the diagnostic criteria for pSS; ② Diagnosed with pSS for the first time; ③Aged from 18 to 67 years old; ④ Those who voluntarily participate in this study; ⑤ The clinical data are complete.

#### 2.1.2 Exclusion criteria

① Do not meet the diagnostic criteria for pSS; ② The patient or their immediate family members have other systemic autoimmune diseases (including Systemic Lupus Erythematosus, Dermatomyositis, Rheumatoid arthritis, Adult-Onset Still’s Disease, etc.); ③ Accompanied by severe cardiovascular, brain, liver, kidney, lung and blood system diseases or tumors; ④ Infectious diseases; ⑤ Patients with mental illness and those without civil capacity; ⑥ Pregnant or lactating women.

### 2.2 Reagents, instruments, and analysis software

Illumina’s NovaSeq 6000 gene sequencer; BioTek’s RNA extraction kit; Thermo’s NanoDrop 2000 ultra-micro spectrophotometer; TaKaRa’s reverse transcription kit; Roche’s LightCycler 480 real-time fluorescent quantitative PCR (RT-qPCR) instrument.

### 2.3 Experimental methods

#### 2.3.1 Preparation of peripheral blood mononuclear cells and neutrophils

Ficoll-Paque Plus (GE Healthcare, USA) was added to the venous blood of the pSS group and HC group to obtain two groups of human PBMCs through density gradient centrifugation. 0.4% trypan blue staining solution was mixed for staining, and cell concentration and viability were evaluated and recorded under a microscope. Cell qualification criteria: cell viability >85%, total number of cells >20,000, impurities or red blood cells accounting for <20%.

#### 2.3.2 Library construction and scRNA-seq

Prepare a PBS single cell suspension of 2.0×10^5^ cells/mL and add Ampure XP purified magnetic beads with unique cell tags (Barcode) and Unique Molecular Identifiers (UMI) to label cells and mRNA. The mRNA captured by magnetic beads is reverse transcribed into cDNA and amplified. A Qubit fluorometer and an Agilent fragment analyzer were used for cDNA concentration detection and fragment size detection, respectively. Using the GEXSCOPE™ Single Cell RNA Library Kit (Singleron), scRNA-seq libraries were constructed according to the Singleron GEXSCOPE™ protocol. Following quality control, the library was sequenced on a NovaSeq™ 6000 sequencer (Illumina) in 2×150bp mode.

### 2.4 scRNA-seq data analysis

Data from a total of 6 samples from 3 pSS patients and 3 HCs were processed using CeleScope^®^ software (https://github.com/singleron-RD/CeleScope; Singleron Biotechnology) with default parameters to generate a gene expression matrix from raw reads. FeatureCounts (v2.0.1) was employed to construct a gene expression matrix, calculate the number of UMIs per gene in each cell, and facilitate subsequent analysis.

### 2.5 Quality control, dimension reduction, clustering, and annotation

Quality control, dimension reduction, and clustering were performed using Scanpy v1.8.2 under Python 3.7 (7). After normalizing the data, the top 2000 variable genes were selected for subsequent Principal Component Analysis (PCA). The top 20 principal components were used for clustering and dimensionality reduction. Cell clusters were visualized using Uniform Manifold Approximation and Projection (UMAP) with the Seurat function RunUMAP. Cell types were annotated using the SynEcoSys™ database (Singleton Biotechnology).

### 2.6 Pseudotime analysis

Cell differentiation trajectories of neutrophils were reconstructed using Monocle2 v 2.10.0 (8). Trajectories were visualized via the “plot_cell_trajectory” function in Monocle2.

### 2.7 Cell-cell interaction analysis

CellphoneDB (v2.1.0) was used to predict the interactions between cells based on known ligand-receptor pairs (9). Visualization was achieved using the heatmap_plot and dot_plot functions within CellphoneDB.

### 2.8 RT-qPCR verification of *LY6E* and *CLEC12A*

#### 2.8.1 Primer sequence synthesis

Human target gene sequences for *LY6E* and *CLEC12A* were retrieved from the Primer Bank. Primer sequences with higher specificity were screened using the BLAST website. Primers were synthesized by Shenzhen BGI Technology Co., Ltd. and Shanghai Sangon Biotech Co., Ltd., respectively.

#### 2.8.2 Total RNA extraction, purity identification, and reverse transcription

Total RNA was extracted from subjects’ peripheral venous blood (≥5 mL) using the Bioteke RNA Extraction Kit RP4002. The absorbance (A) values of RNA samples at 260 nm and 280 nm were measured using a NanoDrop 2000 ultra-micro spectrophotometer (Thermo Fisher Scientific, USA). RNA quality and purity were identified via the A_260/280_ ratio. Samples with a ratio between 1.8 and 2.0 were selected, and their concentration was then calculated. Reverse transcription was performed according to the PrimeScript™ RT Master Mix kit instructions (Takara Bio Inc., Japan). 1 μg of total RNA was transcribed into cDNA.

#### 2.8.3 RT-qPCR

The RT-qPCR method was employed to detect the expression levels of LY6E and CLEC12A. A 20 μL total reaction mixture was prepared as follows: 10 μL of TB Green^®^ Premix Ex Taq™ II (Takara Bio Inc., Japan), 1 μL of forward primer, 1 μL of reverse primer, 2 μL of cDNA, and 6 μL of RNase-free H_2_O. Reaction conditions: 95 °C for 30 seconds, 1 cycle; 95 °C for 5 seconds, 60 °C for 30 seconds, 40 cycles; 95 °C for 5 seconds, 60 °C for 60 seconds, 50 °C for 30 seconds, 1 cycle. Each sample was tested in triplicate wells using a Roche LightCycler 480 PCR instrument. β-actin served as the internal control primer. Target gene Ct values were normalized to those of β-actin Ct values, and relative gene expression was calculated using the 2^-ΔΔCt^ method. The forward primer for the target gene LY6E is 5’-CAGCTCGCTGATGTGCTTCT-3’, and the reverse primer is 5’-CAGACACAGTCACGCAGTAGT-3’; the forward primer for the target gene CLEC12A is 5’-TAGCCACCAAATTATGTCGTGAG-3’, and the reverse primer is 5’-GCTGTCCTTATGCCAAATCCATC-3’; the forward primer for the housekeeping gene is 5’-CCTGGCACCCAGCACAAT-3’, and the reverse primer is 5’-GGGCCGGACTCGTCATAC-3’.

### 2.9 Statistical analysis

Statistical analysis was performed using IBM SPSS Statistics 27 software. Continuous parameters were presented as mean ± SD or median (IQR). For normally distributed continuous data, comparisons between two groups were performed using the unpaired two-tailed Student’s t-test. Comparisons between groups with non-normally distributed data were conducted using the Mann-Whitney U test. The unpaired two-tailed Wilcoxon rank-sum test was performed using R software. GraphPad Prism 10 software, Monocle software, and CellphoneDB software were used for data visualization. A *P*-value < 0.05 was considered statistically significant.

## 3 Results

### 3.1 Relationship Between white blood cell counts and clinical characteristics and disease activity in pSS patients

As critical components of systemic autoimmune inflammation, leukocytes and their subtypes are closely associated with pSS, mediating lymphocyte infiltration and regulating the production of autoantibodies, among other processes. Previous studies have shown that the proportion of Tph cells in peripheral blood, the Tph/Treg ratio, and the platelet/lymphocyte ratio (PLR) are significantly positively correlated with the EULAR Sjögren’s syndrome disease activity index (ESSDAI) score (10, 11). Furthermore, individuals with positive anti-SSA antibodies had significantly lower white blood cell counts than those with negative autoantibodies (*P* < 0.0001) (12). Using WBC 3.5×10^9^/L as the critical value, patients were divided into an elevated WBC group and a decreased WBC group. Neutrophil counts (Z = -3.4384, *P* = 0.0006) and complement C4 levels (t = 2.5062, *P* = 0.0181) in pSS patients showed statistically significant differences in expression between the elevated and decreased WBC groups (**Table 1**).

**Table 1:** Differences between WBC levels and clinical characteristics and disease activity in pSS patients.

Indicators	WBC≥3.5(n=22)	WBC<3.5(n=9)	*t* / *Z* / *X^2^*	*P*
Age	48.68±11.24	44.11±10.02	1.0582	0.2987
ESSDAI score	6.77±4.32	4.89±3.30	1.1718	0.2508
Neutrophil (×109/L)	3.39(2.27,5.30)	1.52(1.18,2.25)	-3.4384	0.0006^*^
ESR (mm/h)	23.85±22.75	29.11±25.26	-0.5572	0.5820
hs-CRP (mg/L)	1.02(0.43,2.66)	0.84(0.34,2.99)	-0.4480	0.6542
IL-6(pg/ml)	3.19(2.03,4.98)	2.79(1.92,7.64)	-0.4445	0.6567
IgG (g/L)	19.46±7.68	20.84±6.64	-0.4685	0.6430
IgA (g/L)	3.15±1.43	3.31±1.39	-0.2913	0.7729
IgM (g/L)	1.09(0.78,1.62)	0.76(0.47,1.18)	-1.4148	0.1571
C3 (g/L)	1.02±0.18	0.89±0.22	1.7348	0.0934
C4 (g/L)	0.23±0.08	0.15±0.06	2.5062	0.0181^*^
Anti-SSA antibody (n%)	20(90.91%)	9(100%)	\	1.0000a
Anti-SSB antibody (n%)	13(59.09%)	6(66.67%)	\	1.0000a
Anti-Ro52 antibody (n%)	17(77.27%)	7(77.78%)	\	1.0000a
LY6E	3.23(2.18,6.48)	4.81(2.61,6.17)	-0.6669	0.5049
CLEC12A	4.86(1.87,9.56)	10.13(1.89,15.04)	-1.4897	0.1363

*P < 0.05, statistically significant. a: Fisher exact test was used.

### 3.2 Clustering analysis results of PBMCs samples from the pSS and HC groups

**Figure 1:**
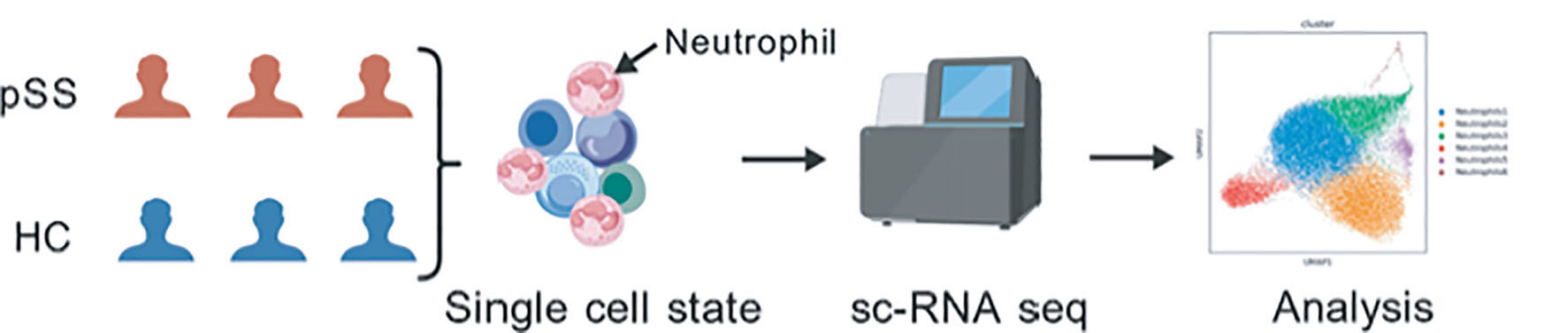
Single-cell transcriptome flow maps of PBMCs samples in the pSS group and the HC group. Overview of study design (created with BioGDP.com): Neutrophil transcriptome data were obtained from pSS (n=3) and HC (n=3) PBMCs samples using the GEXSCOPE™ single-cell RNA sequencing platform.

**Figure 2:**
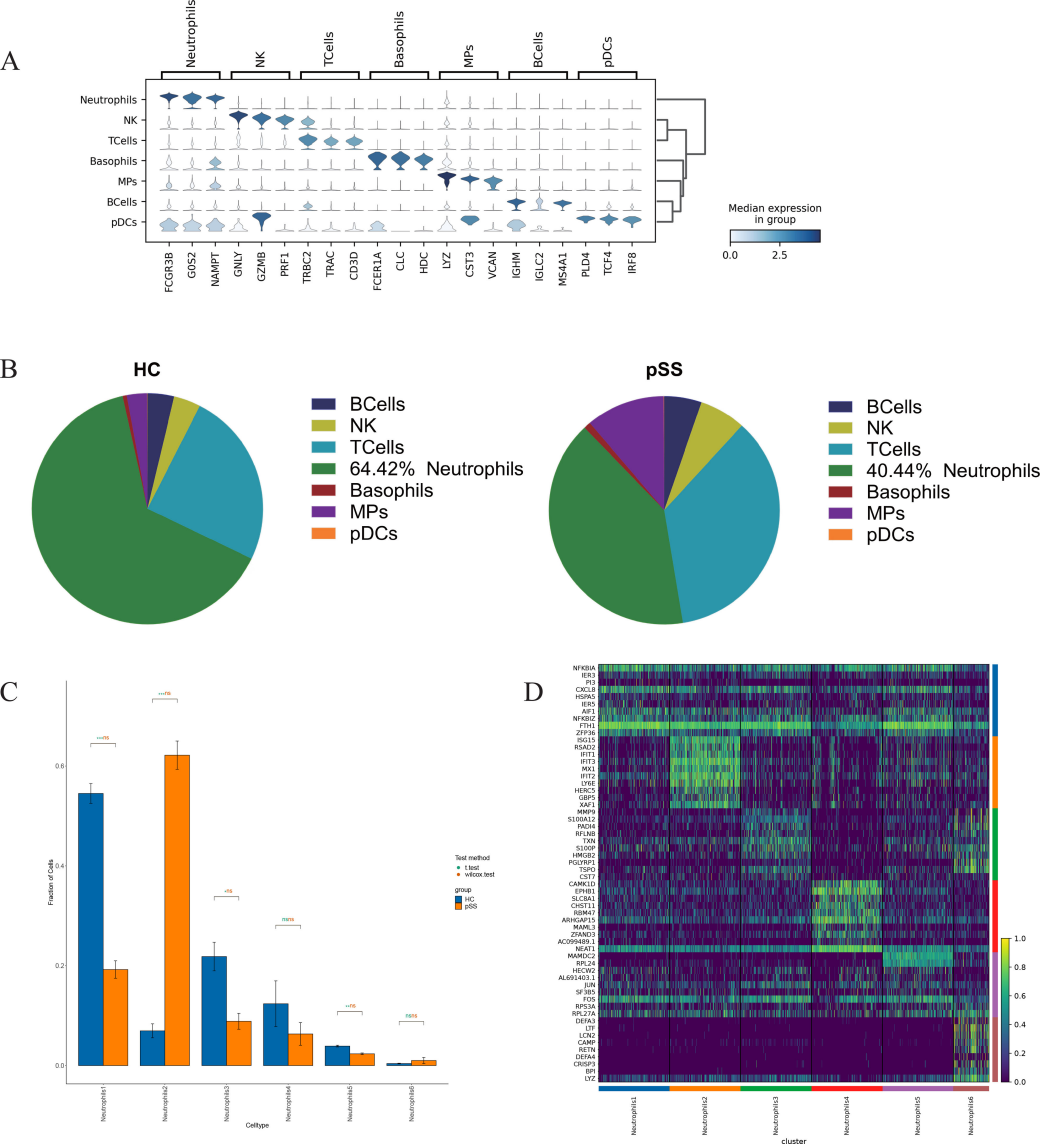
Distribution of PBMCs neutrophils and differentially expressed genes in the pSS group and HC group. **(A)** Violin plot depicts differential gene expression in different immune cells. **(B)** Distribution of each cell type in PBMCs from the HC group and the pSS group. **(C)** Comparison of neutrophil subsets between the HC group and pSS group (Student’s t-test and Wilcoxon rank-sum test). *P < 0.05; **P < 0.01; ***P < 0.001; ns, not significant (*P* ≥ 0.05). **(D)** Heatmap of the top 10 DEGs in neutrophil subsets.

As shown in **Figure 1**, PBMCs were obtained from 3 pSS and 3 HC cases. Single-cell RNA sequencing was performed using the GEXSCOPE™ platform, yielding single-cell transcriptome data from both groups of PBMCs (13). These data were integrated and annotated based on neutrophil marker genes *CSF3R*, *CXCR2*, and *FCGR3B* (14). Seven types of immune cells were identified (**Figure 2A**).

### 3.3 Neutrophil proportion and subsets classification from pSS and HC groups

A total of 33,919 neutrophils were identified in 6 PBMC samples from the pSS and HC groups. Among these, the HC group contained 20,236 neutrophils (64.42%), while the pSS group contained 13,683 neutrophils (40.44%) (**Figure 2B**). Compared with the HC group, the pSS group exhibited lower proportions of Neutrophil 1, Neutrophil 3, Neutrophil 4, and Neutrophil 5 subsets, while showing higher proportions of Neutrophil 2 and Neutrophil 6 subsets (**Figure 2C**). The annotation of neutrophil subsets is based on the differential gene expression profiles among subsets, including Neutrophil 1 (*NFKBIA*, *IER3*, *PI3*), Neutrophil 2 (*ISG15*, *RSAD2*, *IFIT1*), Neutrophil 3 (*MMP9*, *S100A12*, *PADI4*), Neutrophil 4 (*CAMK1D*, *EPHB1*, *SLC8A1*), Neutrophil 5 (*MAMDC2*, *RPL24*, *HECW2*) and Neutrophil 6 (*DEFA3*, *LTF*, *LCN2*); as shown in **Figure 2D**.

### 3.4 Gene ontology enrichment analysis results and Kyoto Encyclopedia of Genes and Genomes enrichment analysis results for DEGs in neutrophil subsets

#### 3.4.1 GO and KEGG enrichment analysis of DEGs of neutrophils 2 subset in the pSS group

**Figure 3:**
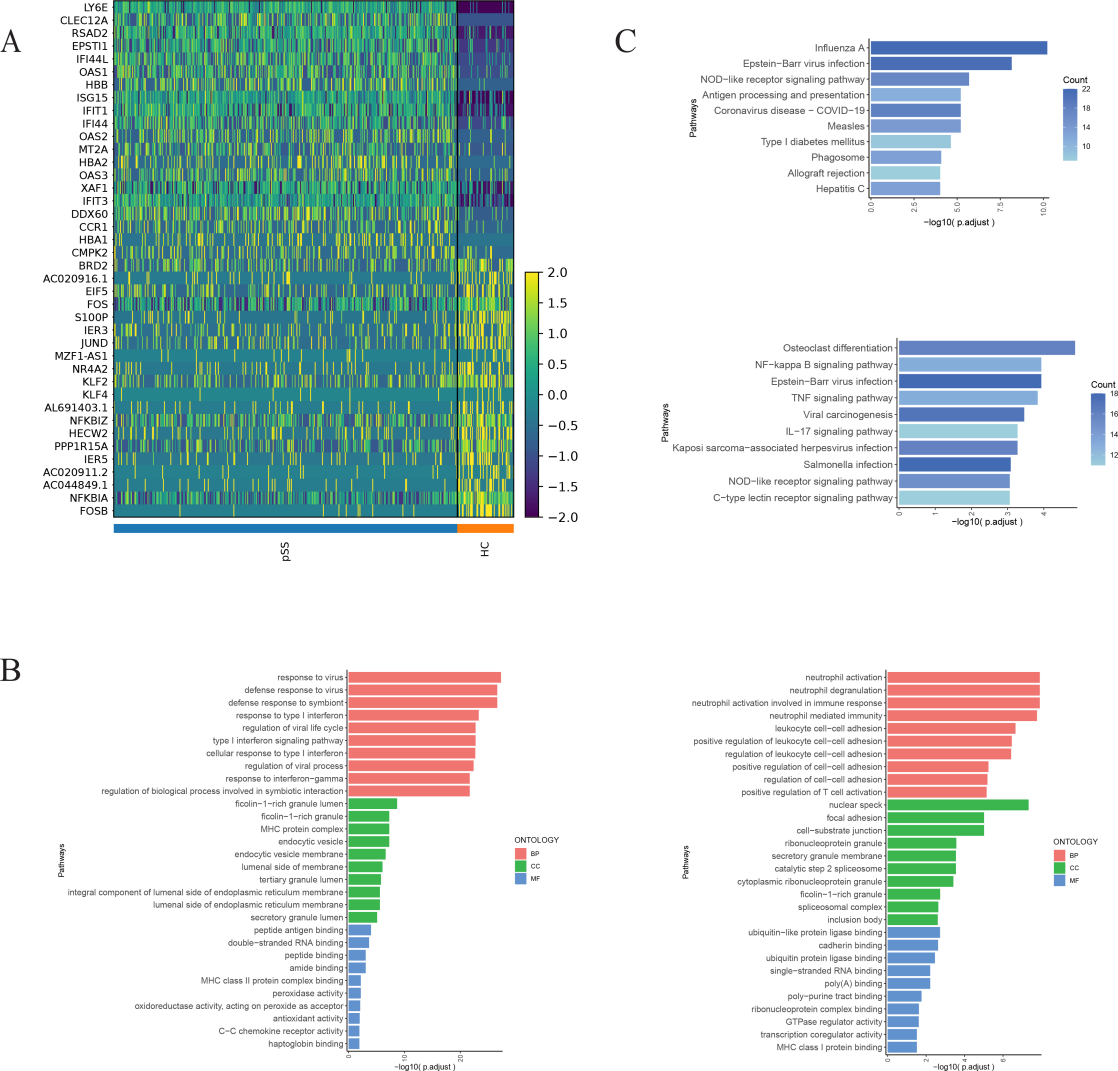
Differential gene enrichment analysis of neutrophil 2 subset in the pSS group. **(A)** Heatmap of the top 20 upregulated and top 20 downregulated genes sorted by score among Neutrophil 2 subset groups between the pSS group and the HC group. **(B)** GO enrichment analysis of upregulated genes in the Neutrophils 2 subset of the pSS group (left), GO enrichment analysis of downregulated genes (right). Pathways are displayed on the vertical axis. The longer the column, the more significant the enrichment effect. **(C)** KEGG enrichment analysis of upregulated genes of Neutrophils 2 subset in pSS group (top), KEGG enrichment analysis of downregulated genes (bottom).

Compared with the HC group, the pSS group exhibited upregulated DEGs in the Neutrophil 2 subset, including *LY6E*, *CLEC12A*, *RSAD2*, *EPSTI1*, *IFI44L*, etc. (**Figure 3A**). GO enrichment analysis revealed that these upregulated genes were primarily enriched in biological processes (BP), such as defense response to virus, regulation of viral life cycle, and response to type I IFN. At the cellular component (CC) level, enrichment occurred in ficolin-1-rich granules, major histocompatibility complex (MHC) protein complexes, and endocytic vesicles. At the molecular function (MF) level, enrichment was observed in peptide binding, double-stranded RNA binding, amide binding, and MHC class II protein complex binding. Downregulated genes primarily include *BRD2*, *AC020916.1*, *EIF5*, *FOS*, *S100P*, etc., predominantly enriched in BP such as neutrophil activation and degranulation, and leukocyte cell-cell adhesion. At the CC level, enrichment was observed in nuclear specks, focal adhesion, cell-substrate junctions, ribonucleoprotein granules, and secretory granule membrane. At the MF level, enrichment was seen in ubiquitin-like protein ligase binding, cadherin binding, single-stranded RNA binding, and poly (A) binding (**Figure 3B**). KEGG analysis revealed that the upregulated DEGs were enriched in pathways including Influenza A, Epstein-Barr virus (EBV) infection, and NOD-like receptor signaling pathway. The downregulated DEGs were enriched in pathways such as Osteoclast differentiation and nuclear factor (NF)-κB signaling pathway (**Figure 3C**).

#### 3.4.2 GO and KEGG enrichment analysis of DEGs of neutrophils 6 subset in the pSS group

**Figure 4:**
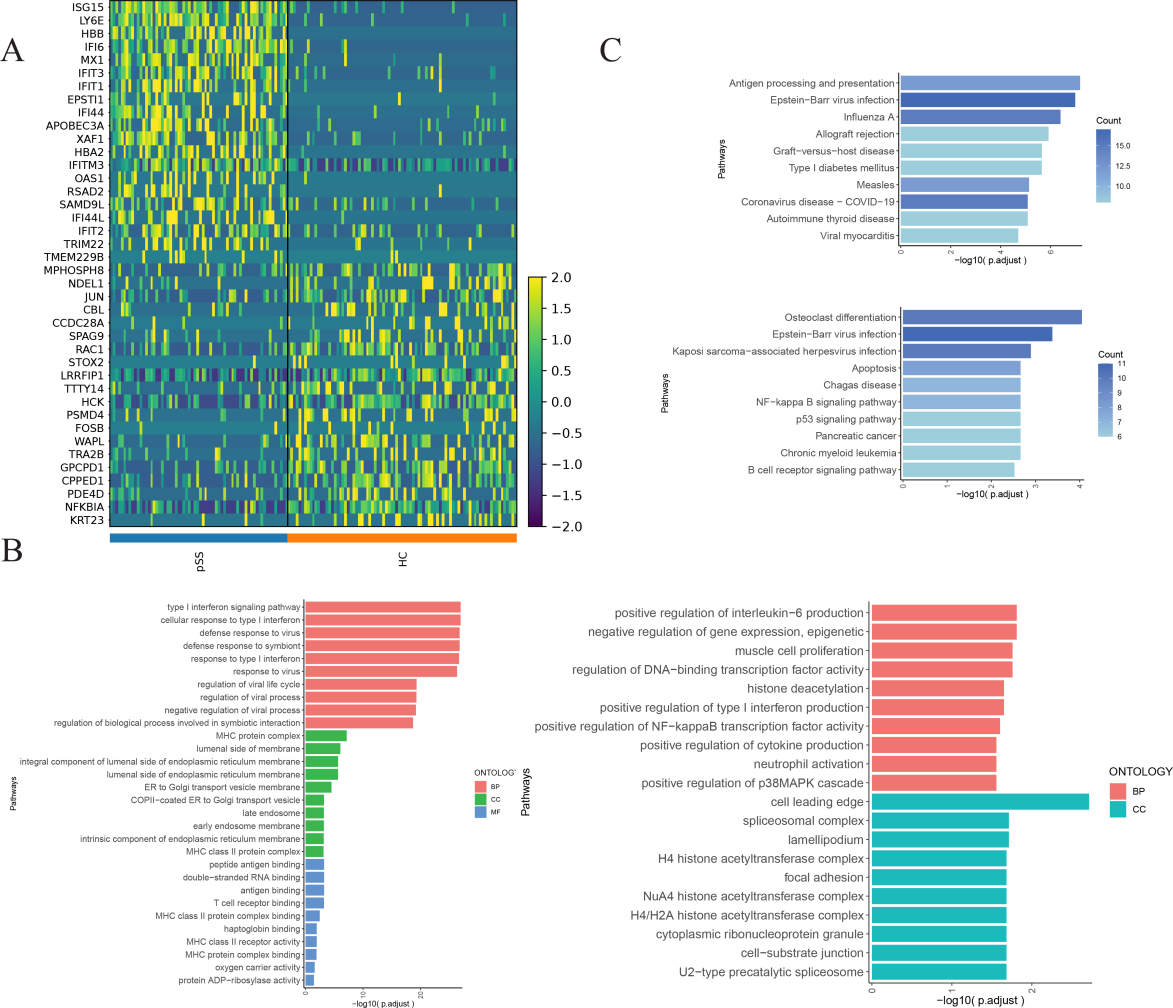
Differential gene enrichment analysis of neutrophil 6 subset in the pSS group. **(A)** Heatmap of the top 20 upregulated and top 20 downregulated genes sorted by score among Neutrophil 6 subset groups between the pSS group and the HC group. **(B)** GO enrichment analysis of upregulated genes in the Neutrophils 6 subset of the pSS group (left), GO enrichment analysis of downregulated genes (right). Pathways are displayed on the vertical axis. The longer the column, the more significant the enrichment effect. **(C)** KEGG enrichment analysis of upregulated genes of Neutrophils 6 subset in pSS group (top), KEGG enrichment analysis of downregulated genes (bottom).

Compared with the HC group, the pSS group exhibited upregulated DEGs in the Neutrophil 6 subset, including *ISG15*, *LY6E*, *HBB*, *IFI6*, *MX1*, and others (**Figure 4A**). GO enrichment analysis revealed that these genes were primarily enriched in BP, such as the type I IFN signaling pathway and defense response to virus. At the CC level, enrichment occurred in MHC protein complexes, endoplasmic reticulum membranes, and Endoplasmic reticulum (ER) to Golgi transport vesicle membranes. At the MF level, enrichment was observed in antigen binding, double-stranded RNA binding, T cell receptor binding, and MHC class II protein complex binding. The downregulated genes primarily include *MPHOSPH8*, *NDEL1*, *JUN*, *CBL*, *CCDC28A*, etc. They were mainly enriched in BP, such as positive regulation of interleukin-6 (IL-6) production, negative regulation of gene expression (at the epigenetic level), muscle cell proliferation, and regulation of DNA-binding transcription factor activity. At the CC level, enrichment was observed in the cell leading edge and spliceosomal complex (**Figure 4B**). KEGG analysis revealed that the upregulated DEGs were enriched in pathways including Antigen processing and presentation, EBV infection, and Influenza A. The downregulated DEGs were concentrated in pathways such as Osteoclast differentiation, EBV infection, and Kaposi sarcoma-associated herpesvirus infection (**Figure 4C**).

#### 3.4.3 GO and KEGG enrichment analysis results of DEGs among neutrophil subsets

**Figure 5:**
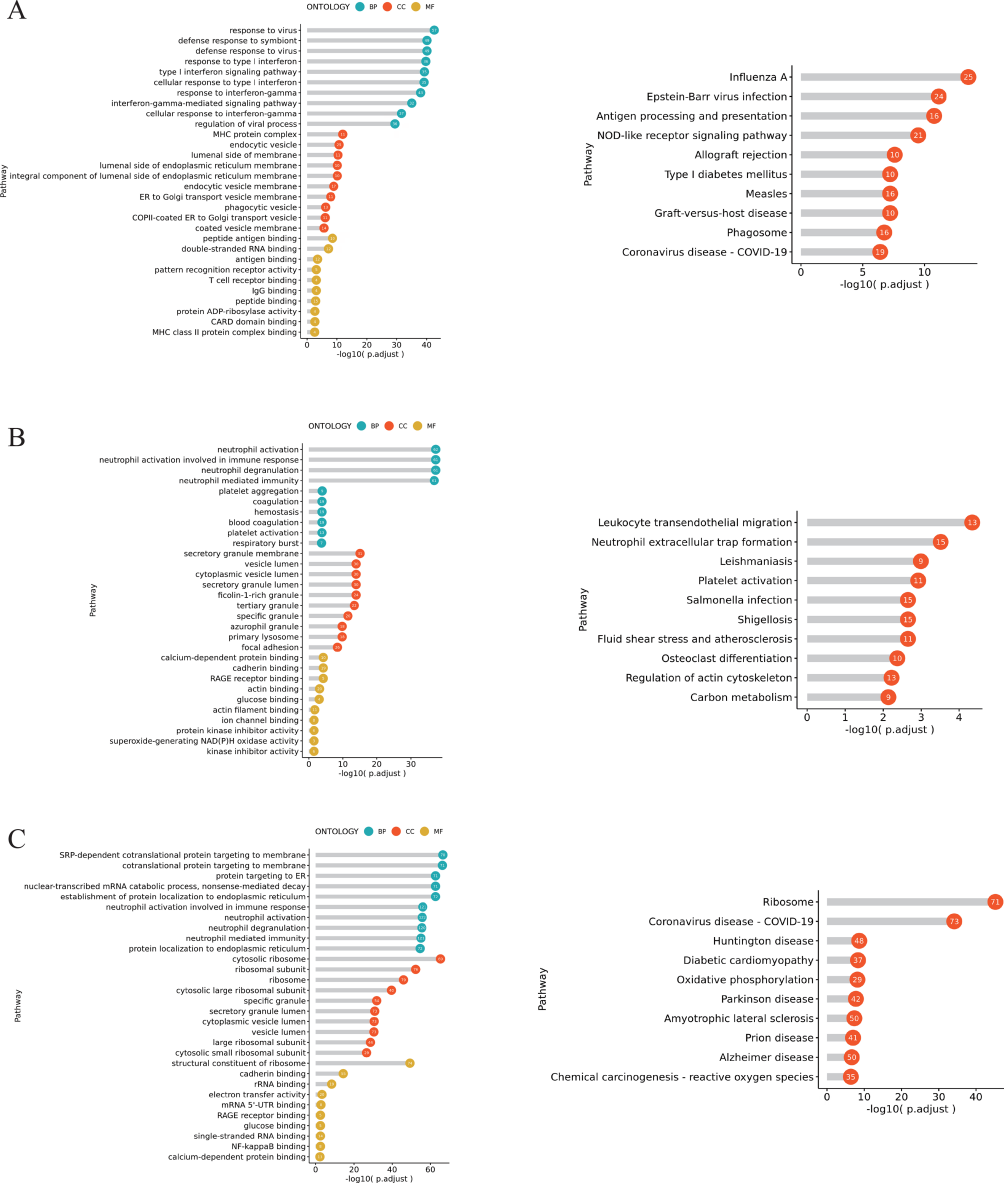
Functional enrichment of DEGs in neutrophil subsets in the pSS group and the HC group. **(A)** GO and KEGG enrichment analysis for the Neutrophils 2 subset. The x-axis represents -log10(p.adjust), while the y-axis shows enriched pathways. Longer lollipop bars indicate more significant enrichment, and numbers within circles denote the number of genes enriched in that pathway. **(B)** GO and KEGG enrichment analysis for the Neutrophils 3 subset. **(C)** GO and KEGG enrichment analysis for the Neutrophils 6 subset.

Enrichment analysis of DEGs across neutrophil subsets is shown in **Figure 5**. Among these, the Neutrophils 2 subset was mainly involved in BP of immune responses to viruses, type I IFN and IFN-γ-mediated cellular responses, as well as KEGG pathways associated with influenza A and EBV infection (**Figure 5A**). The Neutrophils 3 subset showed significant enrichment in BP related to neutrophil-mediated immunity, coagulation and hemostasis mechanisms, as well as KEGG pathways associated with leukocyte transendothelial migration, neutrophil extracellular trap formation, and platelet activation (**Figure 5B**). The Neutrophils 6 subset was significantly enriched in BP related to protein localization and transport, RNA catabolism, and neutrophil-mediated immunity, as well as KEGG pathways associated with ribosomes and Coronavirus disease (COVID-19) (**Figure 5C**).

### 3.5 Gene set variation analysis analysis of neutrophil subsets in the pSS group and HC group

**Figure 6:**
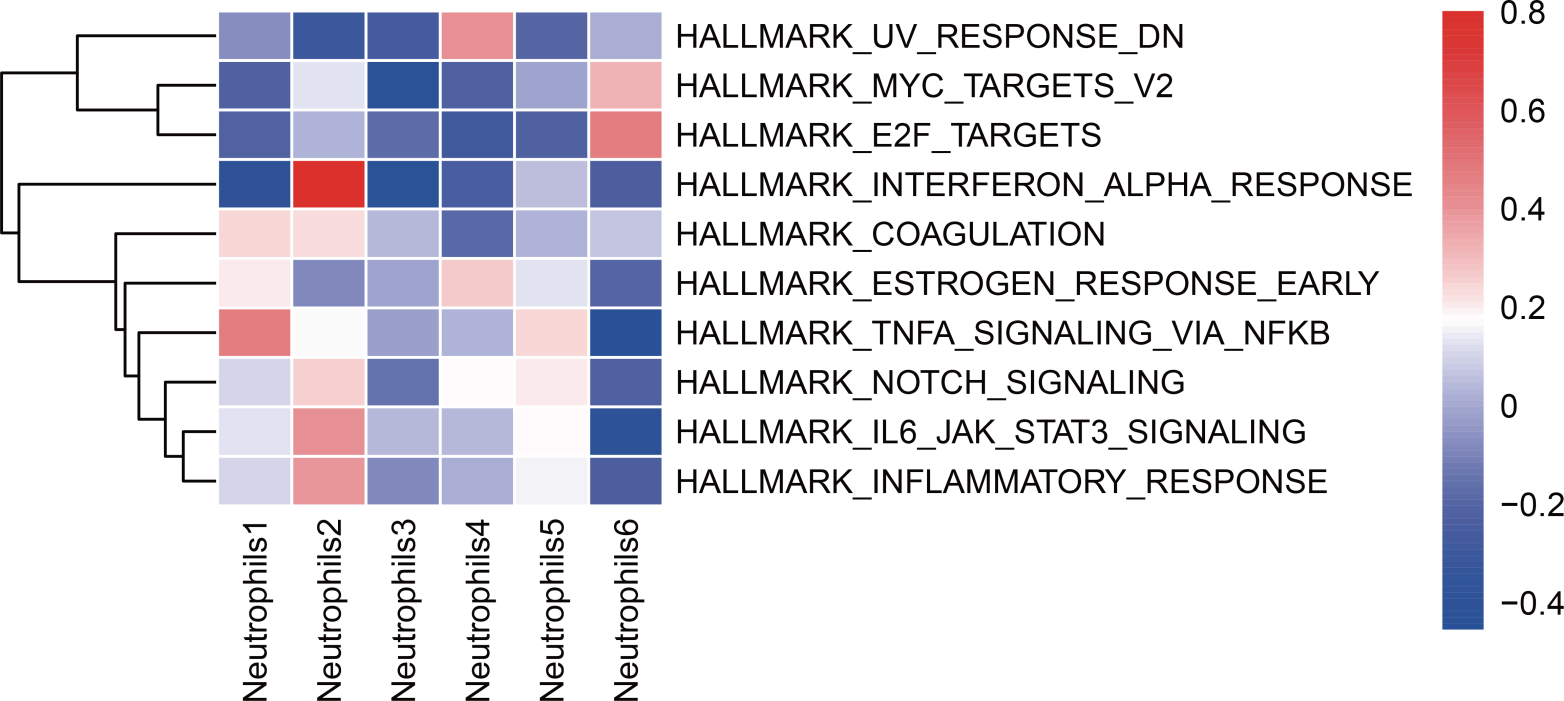
The TOP 10 differential pathways of neutrophil subsets between the pSS group and the HC group. Neutrophil Functional Enrichment Analysis Heatmap Based on the AUCell Algorithm (Differential Pathway GSVA Scores).

Screening analysis identified the top 10 enriched biological pathways in neutrophil subsets between pSS and HC groups, most of which are involved in immune responses and inflammation-related pathways. These pathways included the TNF-α-NF-κB signaling pathway, IFN-α response pathway, IL6-JAK-STAT3 signaling pathway, inflammatory response pathway, UV response pathway, E2F target pathway, among others. GSVA was conducted to validate these findings (**Figure 6**). Notably, the Neutrophils 2 subset activates the IFN-α response pathway most significantly.

### 3.6 Transcription factors in neutrophil subsets from the pSS and HC groups

**Figure 7:**
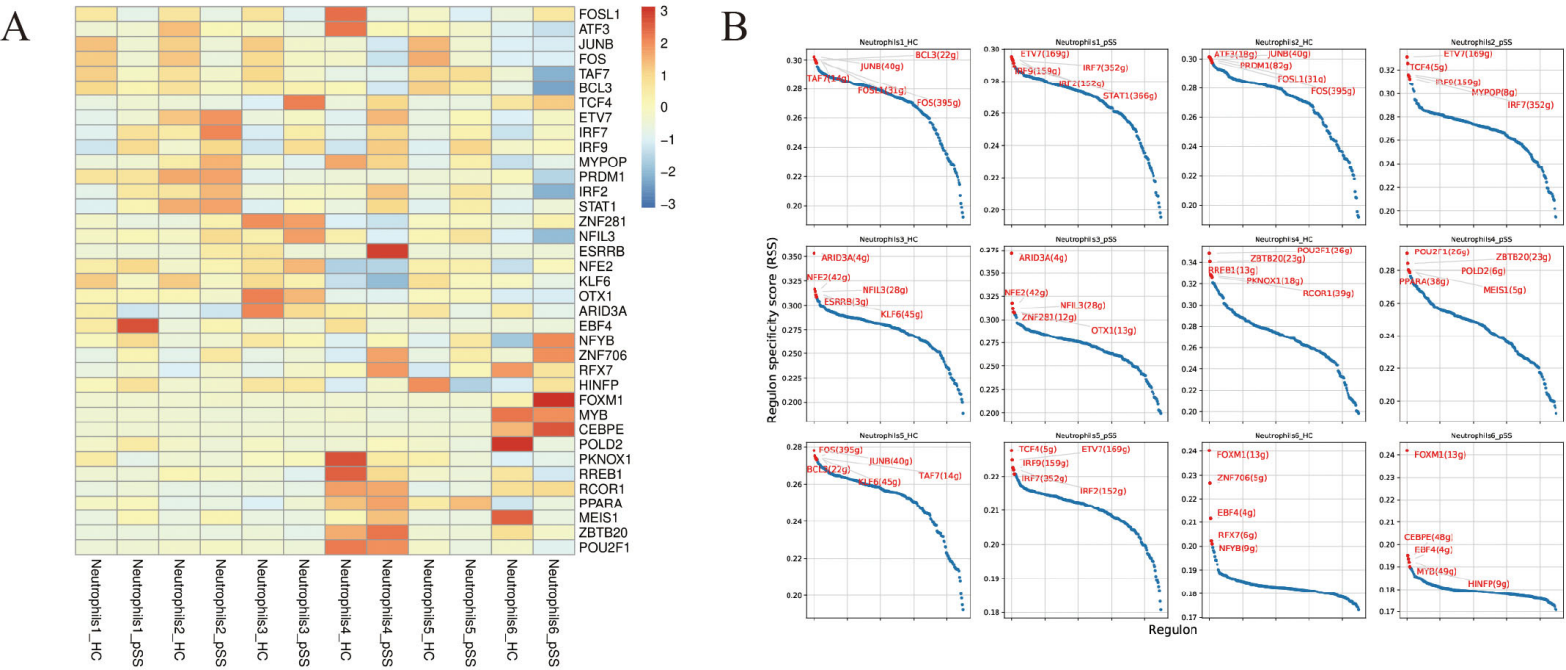
Differential expression of transcription factors in each subset of neutrophils between the pSS group and the HC group. **(A)** Activity of important TFs in neutrophil subsets between the pSS group and HC group, as shown by AUCell heatmap. **(B)** Distribution of regulatory specificity scores (RSS) values across neutrophil subsets in the pSS group and HC group. The ordinate represents the RSS specificity score. Higher RSS values indicate a stronger potential association between the cell subset and specificity.

SCENIC analysis was performed on the pSS group and the HC group (15, 16) (**Figure 7A**). Compared with the HC group, the expression of TF in each neutrophil subset differed in the pSS group. Specifically, the Neutrophil 1 subset had the highest expression of the EBF4, the Neutrophil 4 subset highly expressed the ESRRB, and the Neutrophil 6 subset mainly upregulated the FOX family member FOXM1. TFs such as FOS, JUNB, and BCL3 in the HC group were identified as top regulators based on their regulon specificity scores (RSS), indicating increased activity. Conversely, TFs highly enriched in the pSS group included IRF7, IRF9, and ETV7 (**Figure 7B**).

### 3.7 Interactions between neutrophils and other cells in the pSS group

**Figure 8:**
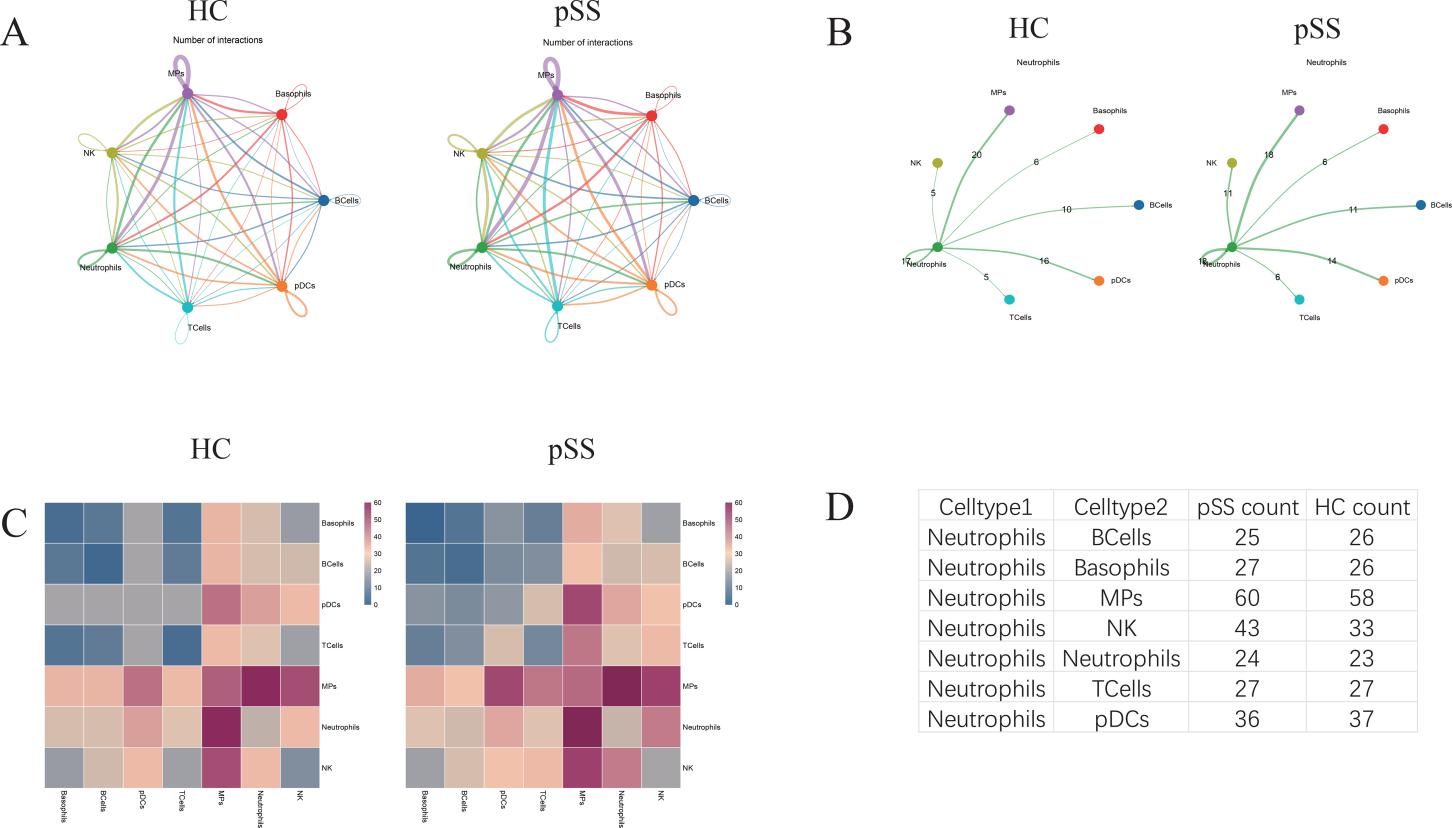
Analysis of the interaction between neutrophils and other cells in the pSS group and the HC group. **(A)** Circle plot illustrating interactions between HC and pSS group cells (removing interaction pairs where both are receptors or both are non-receptors). Line colors denote cell types, while line thickness reflects the quantity and strength of interactions. **(B)** Shell diagram of interactions between HC and pSS group neutrophils acting as ligand cells and other receptor cells (excluding interactions where both partners are receptors or both are non-receptors). Line colors correspond to ligand cell types, and line thickness positively correlates with interaction pair count. **(C)** Heatmap showing the number and strength of interactions between HC and pSS group immune cells. **(D)** Number of interaction pairs between neutrophils and other cell types in the HC and pSS groups.

Neutrophils in the pSS group exhibited enhanced interactions with natural killer (NK) cells, mononuclear phagocytes (MPs), basophils, and other neutrophils compared to the HC group. In both groups, the most numerous interaction pairs were observed between neutrophils and MPs (**Figures 8A-D**).

**Figure 9:**
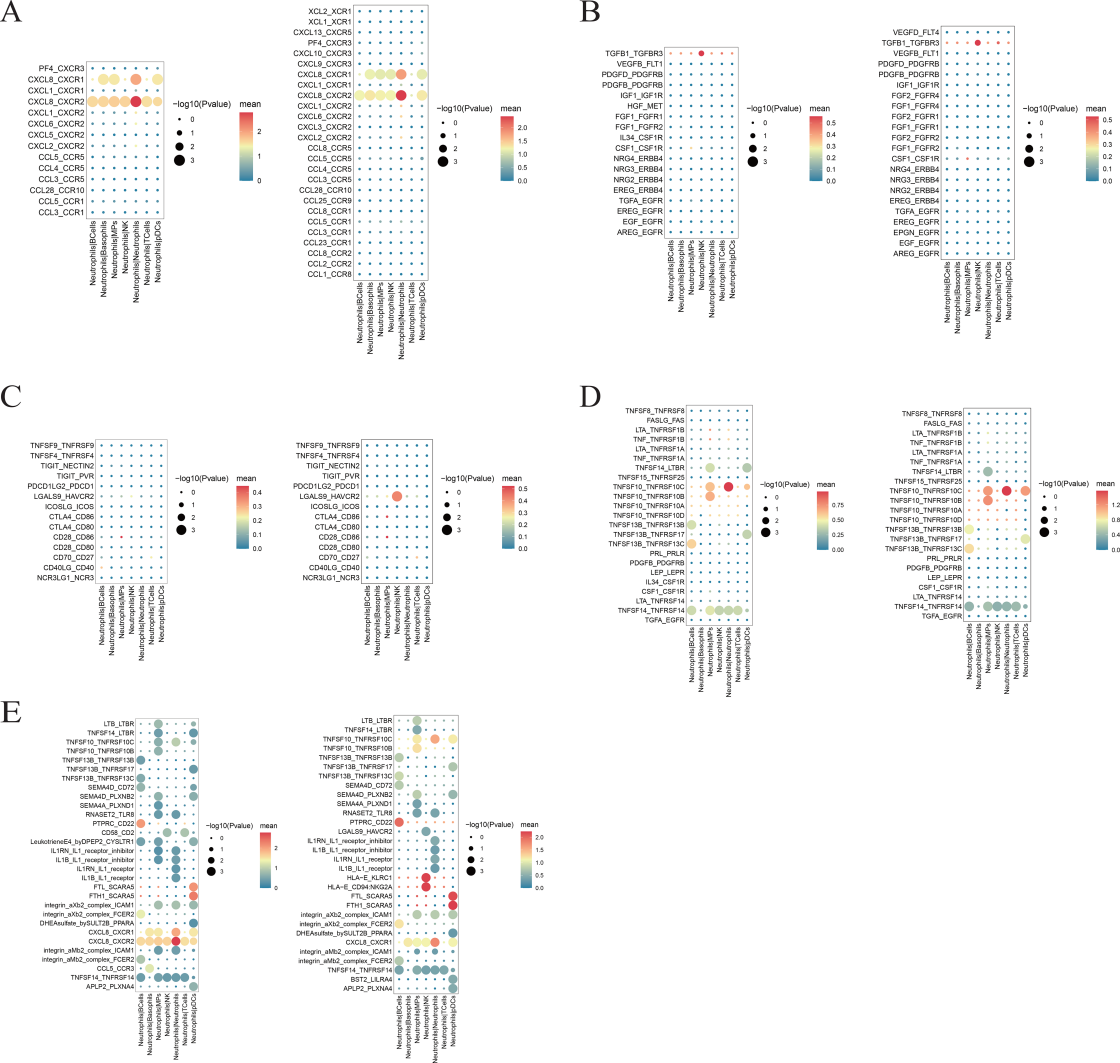
Prediction diagram of interaction pairs between neutrophils in the pSS group and the HC group as ligand cells and other cells. **(A)** Predicted chemokine interaction pairs between neutrophils and other cell types in the HC group (left) and pSS group (right). **(B)** Predicted growth factor interaction pairs between neutrophils and other cell types in the HC group (left) and pSS group (right). **(C)** Predicted immune checkpoint interaction pairs between neutrophils and other cell types in the HC group (left) and pSS group (right). The x-axis denotes cell type pairs, with the cell before the “|” being the ligand cell and the cell after the “|” being the receptor cell; the y-axis denotes interaction gene pairs, with the gene before the “_” being the ligand gene and the gene after the “_” being the receptor gene. Colors indicate the expression mean, and the dot size represents the p-value. **(D)** Predicted cytokine interaction pairs between neutrophils and other cell types in the HC group (left) and pSS group (right). **(E)** Predicted rank 30 interaction pairs between neutrophils and other cell types in the HC group (left) and pSS group (right).

Intercellular interactions are primarily categorized by function into chemokines, growth factors, immune checkpoints, and cytokines. The top 30 most significant interaction pairs are termed the rank 30 interaction pairs. When neutrophils act as ligand cells, pSS group neutrophils exhibit significantly enhanced interaction with NK cells via the LGALS9_HAVCR2 axis compared to the HC group. Regarding the rank 30 interaction pairs, neutrophils in the pSS group exhibited significantly enhanced interactions with plasmacytoid dendritic cells (pDCs) via the FTH1/FTL_SCARA5 axis compared to the HC group (**Figures 9A–E**).

**Figure 10:**
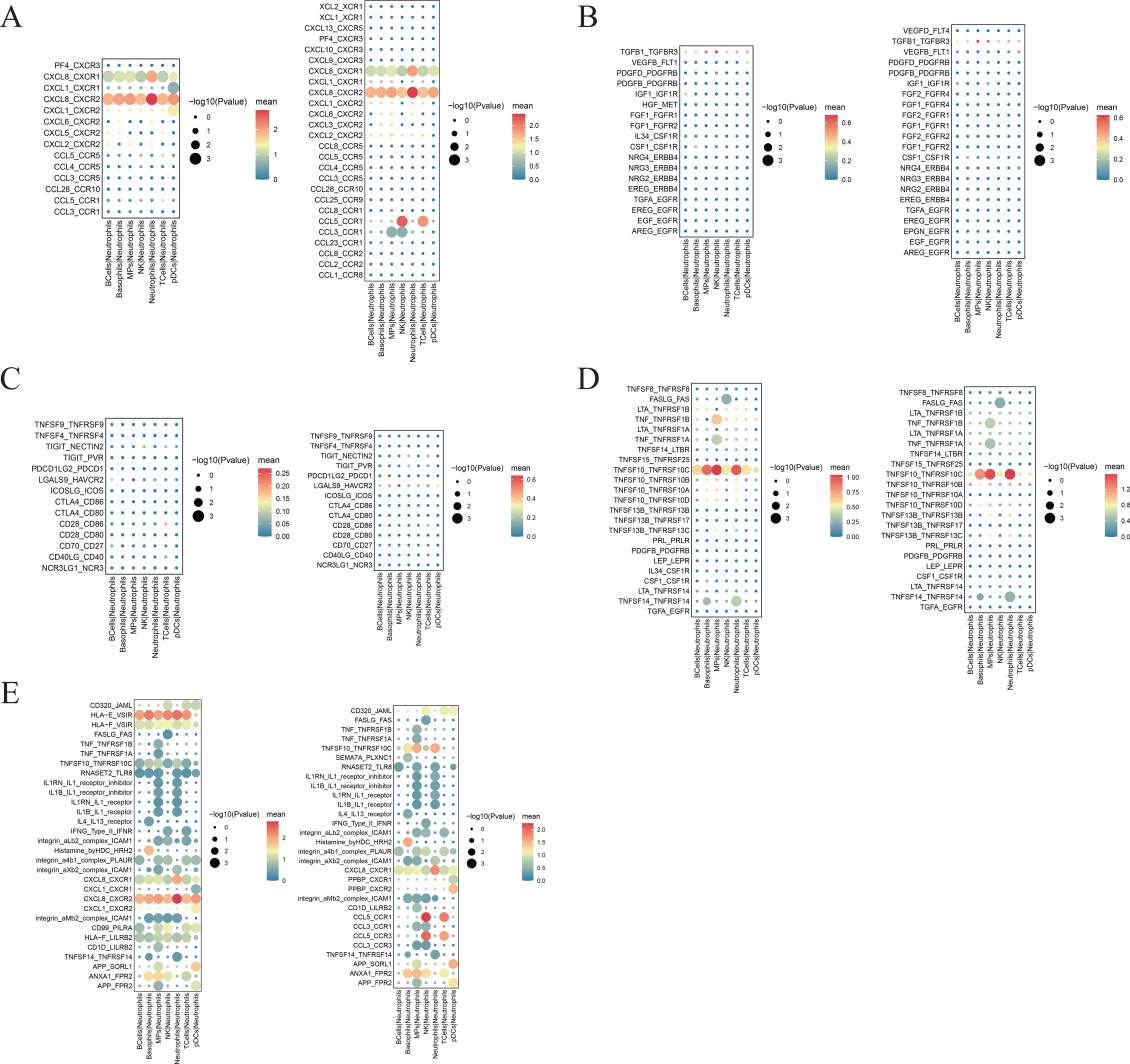
Prediction diagram of interaction pairs between neutrophils in the pSS group and the HC group as receptor cells and other cells. **(A)** Predicted chemokine interaction pairs between neutrophils and other cell types in the HC group (left) and pSS group (right). **(B)** Predicted growth factor interaction pairs between neutrophils and other cell types in the HC group (left) and pSS group (right). **(C)** Predicted immune checkpoint interaction pairs between neutrophils and other cell types in the HC group (left) and pSS group (right). The x-axis denotes cell type pairs, with the cell before the “|” being the ligand cell and the cell after the “|” being the receptor cell; the y-axis denotes interaction gene pairs, with the gene before the “_” being the ligand gene and the gene after the “_” being the receptor gene. Colors indicate the expression mean, and the dot size represents the p-value. **(D)** Predicted cytokine interaction pairs between neutrophils and other cell types in the HC group (left) and pSS group (right). **(E)** Predicted rank 30 interaction pairs between neutrophils and other cell types in the HC group (left) and pSS group (right).

The CCL5–CCR1 axis significantly enhanced signaling from NK cells to neutrophils in pSS compared to HC when neutrophils serve as receptor cells (**Figures 10A–E**).

### 3.8 Pseudotime series analysis of neutrophils in the pSS group

**Figure 11:**
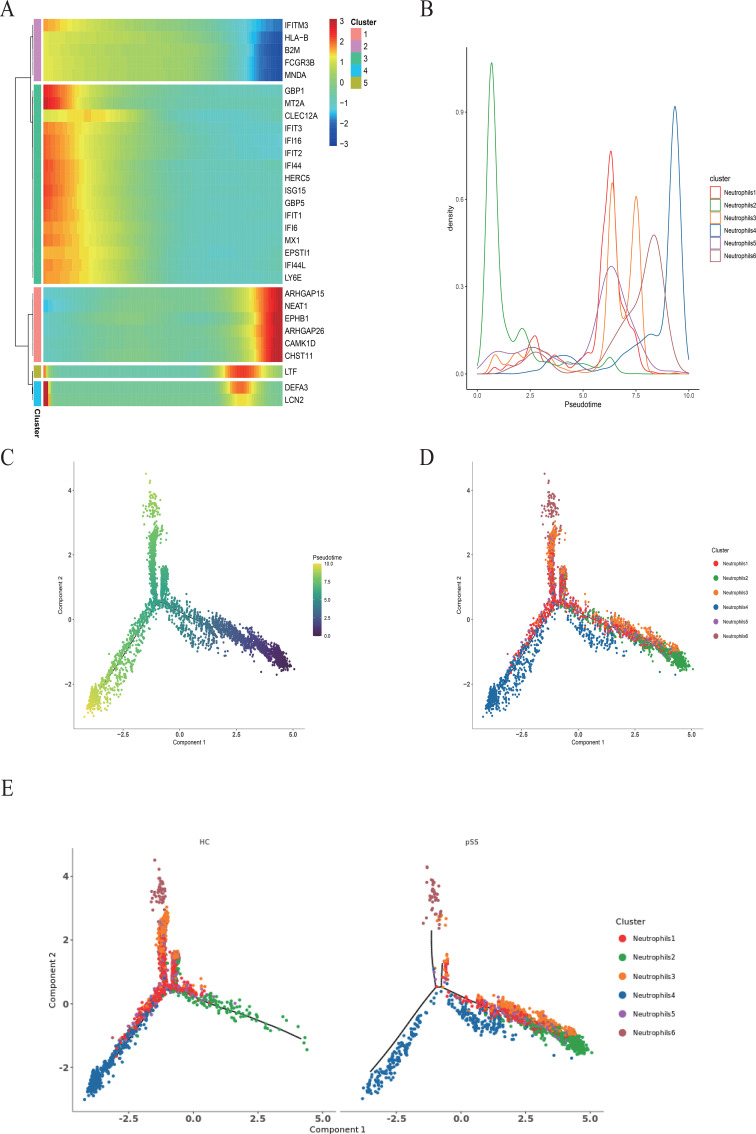
Pseudotemporal analysis of each neutrophil subset in the pSS group. **(A)** Heatmap of pseudo-chronological target gene expression levels, with the x-axis displaying pseudo-time from small to large and the y-axis showing gene expression levels. **(B)** The plot of cell density along the horizontal timeline axis is the pseudotimeline, and the vertical axis represents the density of cell numbers at different time points (displayed for all cells analyzed by all parameters). Higher peaks indicate greater density. Different colors indicate the density distribution of distinct neutrophil subsets over pseudo-time. **(C)** Pseudo-time-ordered plot of all neutrophils, in which the temporal sequence represents the pseudo-chronological sequence of differentiation. **(D)** Colored plot of pseudo-time analysis by neutrophil subset. Different colors denote distinct cell subsets, illustrating differentiation relationships (pseudo-time sequence) among neutrophil subsets. **(E)** Distribution of the HC group (left) and pSS group (right) on pseudo-time trajectories, where different colors denote neutrophil subsets within each group.

Monocle2 was used to construct a neutrophil differentiation trajectory. During neutrophil differentiation, genes were clustered in pseudo-time sequence order into Cluster 1, Cluster 2, Cluster 3, Cluster 4, and Cluster 5. Among them, the expression levels of genes related to cell signaling and cell behavior regulation, such as *ARHGAP15*, *NEAT1*, *EPHB1*, *ARHGAP26*, *CAMK1D*, and *CHST11*, in Cluster 1 were gradually increased. In Cluster 2, the expression levels of genes related to the body’s immune defense response, such as *IFITM3*, *HLA-B*, *B2M*, *FCGR3B*, and *MNDA*, were gradually reduced. Cluster 3 includes 8 IFN-related genes: *ISG15*, *IFIT1*, *IFIT2*, *IFIT3*, *IFI6*, *IFI16*, *IFI44*, and *IFI44L*, as well as *CLEC12A* and *LY6E*, all of which are progressively down-regulated. In Cluster 4, some of the genes involved in bacterial and viral defense responses and neutrophil-mediated immunity, such as *DEFA3* and *LCN2*, were rapidly down-regulated (17). In Cluster 5, the gene *LTF* with antifungal, antibacterial, and anticancer properties underwent a process from up-regulation to down-regulation twice (18) (**Figure 11A**).

### 3.9 Expression of *LY6E* and *CLEC12A* in peripheral blood of pSS group

**Figure 12:**
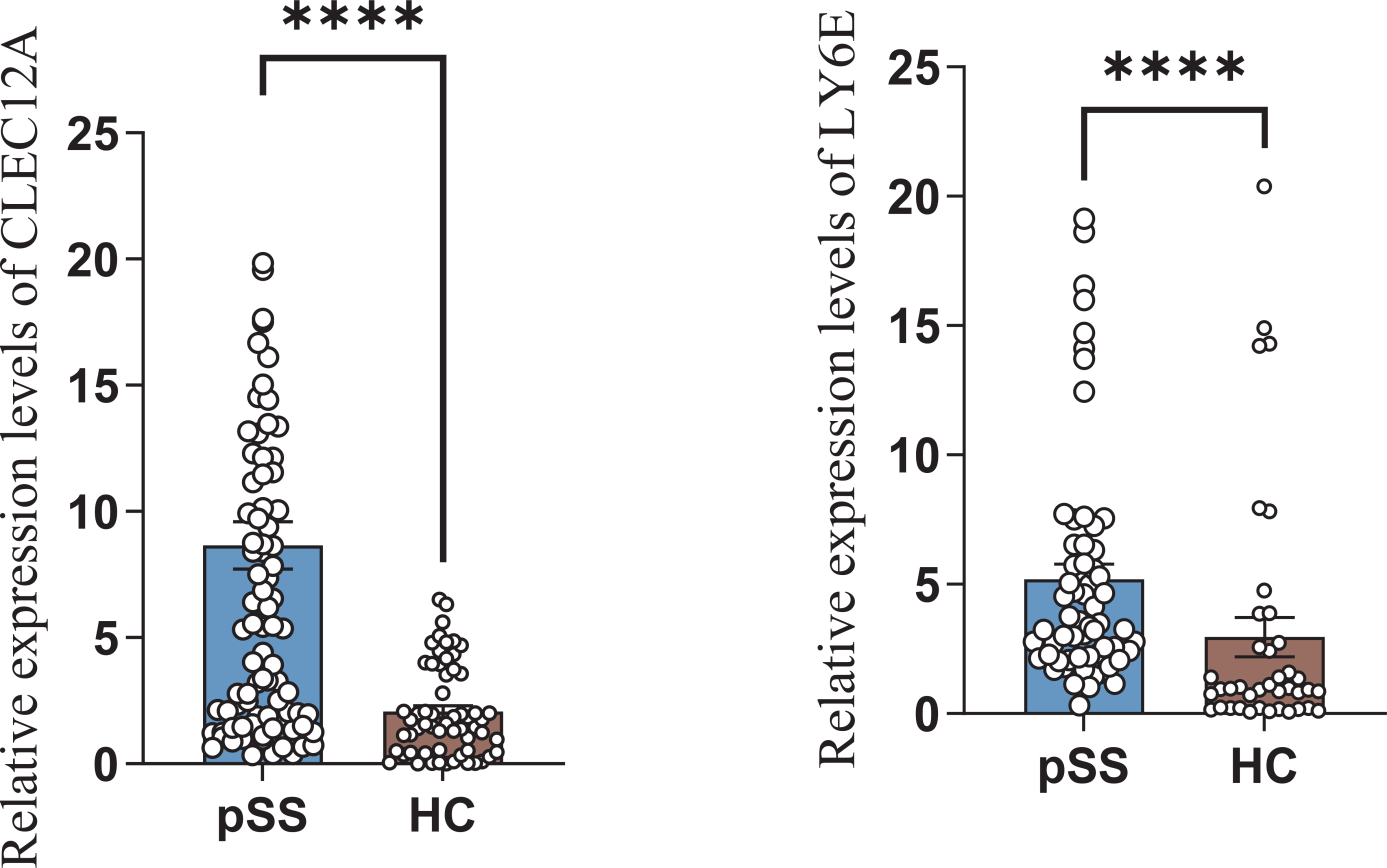
Differential expression of *LY6E* and *CLEC12A* in the pSS group and HC group. **** P < 0.0001.

RT-qPCR further confirmed that compared with the HC group, the mRNA expression of *LY6E* and *CLEC12A* genes in the pSS group was upregulated (**Figure 12**).

## 4 Discussion

The histological features of pSS are varying degrees of lymphocyte infiltration in both salivary glands and lacrimal glands. In recent years, research on pSS has primarily focused on the role of lymphocytes or monocytes, while there are few studies on the role of neutrophils in pSS. Neutrophils constitute the predominant white blood cell population and form the immune system’s first line of defense against bacterial and fungal infections.

The results of this study indicate a significant reduction in total neutrophil counts in PBMCs from the pSS group, consistent with previous research (5, 19). Neutrophils in both the HC and pSS groups were classified into 6 subsets: Neutrophil 1, Neutrophil 2, Neutrophil 3, Neutrophil 4, Neutrophil 5, and Neutrophil 6. Comparison of the proportions of neutrophil subsets in pSS and HC showed that the median proportions of neutrophil 2 (60.70% vs 6.71%) and neutrophil 6 (0.43% vs 0.49%) were numerically higher in the pSS group, although these differences did not reach statistical significance. The Neutrophil 1 subset exhibits high expression of IL-17, TNF-α, and Toll-like receptors (TLR) signaling pathways (**Supplementary Figure 5A**). The Neutrophil 2 subset highly expresses interferon-stimulated genes (ISGs) (*ISG15, RSAD2, IFIT1*), suggesting this subset possesses potent antiviral defense responses in the early stages of disease (20). It is speculated that the overexpression of IFN-characteristic genes in the Neutrophil 2 subset holds significant implications for the pathogenesis and progression of pSS. The biological functions of the Neutrophil 3 subset may be mediated through interactions between neutrophils and platelets. Platelets stimulate neutrophil signaling via the secretion of surface receptors and soluble factors, thereby promoting neutrophil recruitment and extravasation to inflammatory sites, release of proinflammatory mediators, oxidative burst, and formation of NETs (21). The Neutrophil 4 subset primarily participates in processes such as signal transduction regulation and molecular modification (**Supplementary Figure S5B**). The Neutrophil 5 subset is primarily involved in processes related to protein localization and transport, as well as viral gene expression (**Supplementary Figure S5C**). The Neutrophil 6 subset, like the Neutrophil 3 subset, is highly expressed in neutrophil-mediated immunity, and like the Neutrophil 5 subset, is highly expressed in protein localization and transport, as well as RNA metabolism.

Previous studies have identified distinct neutrophil subsets. Cerezo-Wallis et al. performed scRNA-seq on neutrophils from various physiological and pathological states, identifying seven distinct transcriptional hubs within NeuMap (22). The IFN-response hub, characterized by high *IFIT1* expression, shows a high degree of consistency with the Neutrophil 2 subset in terms of interferon response signaling and antiviral response characteristics; both are similar to the G5b state (high expression of ISGs, such as *ISG15*, *IFIT3*, *IFIT1*, and *RSAD2*) reported in mice and humans during infection (23). In addition, the immuno-silent hub resembles the Neutrophil 2 and Neutrophil 6 subsets, as both express *CD52*. The IS-I hub matches the gene expression profiles of the Neutrophil 2, Neutrophil 4, and Neutrophil 6 subsets, while the PreNeu hub partially matches the Neutrophil 6 subset and the antigen (Ag) presentation hub partially matches the Neutrophil 1 subset. Analysis of transcriptional dynamics in NeuMap revealed that binding sites associated with the TFs IRF and STAT are primarily enriched in the IFN-response hub. Interferon-responsive neutrophils play a dominant role in infection, inflammation, and ischemia. Specific activation of ISGs (e.g., *IFIT1*, *IFIT3*, *LY6E*, *ISG15*) drives the transition of the immature hub and immuno-silent hub toward the IFN-response hub.

This study found that there is significant functional remodeling of neutrophil subsets in female pSS patients, with Neutrophil 2 exhibiting the most pronounced IFN response characteristics, manifested by high expression of multiple ISGs and accompanied by significant upregulation of IRF7. These results suggest that in pSS, neutrophil abnormalities are not merely reflected in their numbers or overall activation levels, but more likely manifest as the selective expansion of specific functional subsets, with Neutrophil 2 representing a characteristic neutrophil subset driven by interferon signaling. This finding is consistent with observations by Wang et al. in other immune-mediated diseases (24). The study found that female patients with Behçet’s uveitis had higher proportions of IFN-α-responsive neutrophils and T cell-regulatory neutrophils. Among these, IFN-α-responsive neutrophils highly expressed ISGs such as *IFIT2*, *IFIT3*, *ISG15*, and *IRF9*, and were regulated by IRF3, IRF7, and PRDM1. Based on the findings of this study, Neutrophil 2 exhibits a high degree of transcriptional similarity to this class of IFN-α-responsive neutrophils, both demonstrating significant IFN-driven characteristics. It should be noted that within the neutrophil subset framework established in this study, Neutrophil 2 primarily corresponds to the IFN response program, whereas features such as inflammatory degranulation, oxidative burst, and a tendency toward NETosis are more commonly observed in Neutrophil 3. This suggests that neutrophil abnormalities in pSS are not dominated by a single subset but rather represent a remodeling process involving multiple functional subsets. Thus, changes in neutrophil composition in female-predominant autoimmune diseases may share certain commonalities; in pSS, this commonality is particularly manifested as an enhancement of the IFN-responsive neutrophil subset. In addition, the Ifit1+ neutrophils reported by Lu et al. in a mouse model of acute liver failure bear a strong resemblance to Neutrophil 2 in this study (25). This subset significantly overexpresses ISGs such as Ifit1, Rsad2, and Isg15, and is enriched in pathways related to immune responses, leukocyte chemotaxis, cytokine secretion, and IFN-β responses. More importantly, that study confirmed that IRF7 is a key transcriptional regulator driving the differentiation of Ifit1+ neutrophils. Consistent with this, we also observed significantly elevated IRF7 levels in Neutrophil 2 from pSS patients, suggesting that the IRF7-mediated IFN response program may not be specific to a single disease but rather represents a mechanism of neutrophil functional remodeling shared across different inflammatory diseases. In other words, the Neutrophil 2 identified in this study is not only a characteristic subset in pSS but may also represent an interferon-driven neutrophil state that recurs across various disease contexts.

This study demonstrates that scRNA-seq revealed significantly upregulated DEGs in the pSS group compared to the HC group: *CLEC12A*, *LY6E*, *ISG15*, *IFIT1*, and *XAF1*. *LY6E*, one of the type I IFN response genes, encodes a glycosylphosphatidylinositol (GPI)-anchored cell surface protein and plays an important role in cell signal transduction, immune regulation, negative regulation of viral processes, tumor metastasis, and cell adhesion (26, 27). Liu et al. reported that *LY6E* is a diagnostic gene for pSS. This study demonstrates significantly elevated peripheral blood *LY6E* levels in the pSS group, consistent with prior research (26) and suggesting that peripheral blood *LY6E* levels may hold significant clinical importance in pSS patients. *CLEC12A* encodes an inhibitory C-type lectin family receptor that effectively suppresses granulocyte and monocyte/macrophage function, acting as a negative regulator of inflammation (28). In this study, the high expression of the *CLEC12A* gene in neutrophils from the pSS group can suppress neutrophil activation, thereby inhibiting autoimmune responses and preventing excessive inflammation during pSS disease. However, this also increases susceptibility to invasive infections (29). Further validation via RT-qPCR confirmed the high mRNA expression of the *LY6E* and *CLEC12A* genes in the pSS group. This suggests functional commonalities exist among neutrophil subsets rather than independent existence.

GO analysis of upregulated DEGs revealed that, compared to the HC group, BP in neutrophil subsets within the pSS group were enriched in the responses to type I or II interferons and the defense response to virus; CC were enriched in MHC protein complexes, ficolin-1-rich granules, and endoplasmic reticulum membranes; and MF were enriched in antigen binding and MHC class II protein complex binding. KEGG enrichment analysis revealed that compared to the HC group, viral infection pathways including Influenza A, EBV infection, measles, and COVID-19 were significantly activated in neutrophil subsets within the pSS group (**Supplementary Figures S1**–**S4**). Liu et al. highlighted that influenza virus and EBV infection play a central role in the pathogenesis of pSS, inducing autoimmunity through distinct mechanisms (26). Notably, Neutrophil subsets 2 and 4 localize to the secretory granule lumen rather than the ER-to-Golgi transport vesicle membrane, unlike other subsets. ER-to-Golgi transport vesicle membranes are regulated by signaling pathways and feedback regulation of transported substances within the vesicles, suggesting that the protein secretion process of this subset is weaker than that of other subsets (30). The NOD-like receptor signaling pathway showed no significant activation only in the Neutrophil 6 subset. As this pathway participates in antibacterial and antiviral immune responses (31), this indicates that the Neutrophil 6 subset does not participate in antiviral reactions. In contrast, GO analysis of downregulated DEGs revealed that compared to the HC group, BP in Neutrophil 1–5 subsets of the pSS group were concentrated in neutrophil activation, degranulation, and immune responses mediated by these processes. Meanwhile, the Neutrophil 6 subset was primarily enriched in the positive regulation of IL-6 and type I IFN production, suggesting this subset may reduce pSS inflammatory activity (32). KEGG enrichment analysis revealed that, compared to the HC group, neutrophil subsets in the pSS group were significantly enriched in Osteoclast differentiation, IL-17 signaling pathways, and TNF signaling pathways. Notably, the Neutrophil 6 subset specifically downregulated NF-κB signaling pathways and p53 signaling pathways. Further application of GSVA in this study confirmed that the IFN-α response pathway was elevated exclusively in the Neutrophil 2 subset. Research by Nordmark et al. demonstrated that gene expression profiles in PBMCs and minor salivary glands from the pSS group showed upregulation of IFN-I-induced genes, i.e., an “IFN signature” (33). IFN-α promotes autoimmune processes through a vicious cycle mechanism, increasing the production of autoantibodies and generating more endogenous IFN inducers (34). This further suggests the critical role of the Neutrophil 2 subset in pSS.

In the two-dimensional movement trajectories of neutrophil subsets in this study, the Neutrophil 2 subset showed a significant increase in the early phase but a sharp decline in the late phase. The remaining subsets decreased in the early phase and increased in the late phase, with Neutrophil 4 showing a particularly marked elevation in the late phase of pSS (**Figures 11B–D**). Compared to the HC group, the pSS group exhibited increased numbers of Neutrophil 2 and was primarily concentrated in the early disease phase. Neutrophil 4 subsets decreased markedly in the late phase but increased in the mid-phase (**Figure 11E**). The above findings suggest that the Neutrophil 2 subset may undergo active proliferation during the early stages of pSS disease, revealing its critical role at this phase. Pseudo-time analysis indicates that as neutrophils in the pSS group progress from early to late differentiation stages, the body’s immune response, the defense response against pathogenic bacteria, and expression of IFN-related genes are progressively suppressed, while cellular signaling and behavioral regulation capabilities gradually enhance.

This study revealed through TF regulation analysis that EBF4 (Olf-1/EBF-Like 4) is specifically upregulated in the Neutrophil 1 subset in the pSS group. This transcription factor is also highly expressed in human cytotoxic NK cells and CD8+ T cells. EBF4 promotes Fas-mediated apoptosis and regulates molecules crucial for NK cell and CD8+ T cell development, such as TBX21 and EOMES, as well as granzyme and perforin, which are important for immune cytotoxicity (35). This reveals the importance of EBF4 in immune system regulation, but its role in pSS requires further investigation. ESRRB, highly expressed in the Neutrophil 4 subset, maintains cellular pluripotency and influences cell proliferation. It promotes cell proliferation by inhibiting the TGF-β pathway, thereby stimulating cancer progression (36). FOXM1, highly expressed in the neutrophil 6 subset, is a key regulator of cell proliferation, not only modulating cancer cell growth but also enabling evasion of tumor suppression mechanisms (37). Notably, the pSS group in this study showed high enrichment of TFs IRF7, IRF9, and ETV7. IRF7 is the main TF that produces type I IFN and regulates the innate immune response, enhancing the body’s antiviral immune response (38). In autoimmune diseases including ankylosing spondylitis (AS), IRF7 activated by NETs-RNA inhibits CD4^+^ regulatory T cell (Treg) differentiation, exacerbating inflammatory responses (39). Tregs suppress immune responses in pSS, thereby maintaining homeostasis and immune tolerance (40). IRF9 is a key factor in the JAK-STAT pathway that triggers the antiproliferative activity of IFN-α (41). It plays a vital role in antiviral and antitumor immune responses, as well as in maintaining immune homeostasis. ETV7 expression is negatively correlated with progression in various human cancers and responsiveness to immune checkpoint blockade, playing a decisive role in driving terminal exhaustion of CD8 T cells (42).

The communication network between neutrophil subsets and other cells in this study reveals that pSS neutrophils interact closely with NK cells and pDCs via ligand-receptor pairs such as LGALS9_HAVCR2, FTH1/FTL_SCARA5, and CCL5_CCR1. Activated neutrophils exhibit molecular alterations that prolong their lifespan compared to quiescent neutrophils, thereby acquiring many molecular properties of macrophages. Macrophages have been demonstrated to synthesize and secrete chemical attractants CXCL1/CXCL2 to attract neutrophils during tissue infection or injury (43). NK cells are innate lymphocytes with the ability to secrete cytokines and natural cytotoxicity (44). When neutrophils act as ligand cells, the strong interaction between pSS neutrophils and NK cells, compared with the HC group, is mediated by LGALS9_HAVCR2—a key ligand-receptor pair that contributes to the immunosuppressive microenvironment and is a potential target for immunotherapy (45). Compared to the HC group, the enhanced strong interactions between neutrophils and pDCs in the pSS group were primarily associated with increased binding of FTH1/FTL and SCARA5. The ferritin complex formed by FTH1 and FTL helps regulate systemic iron balance and prevents damage caused by iron overload. As a carcinogenic virus, EBV can reshape intracellular iron metabolism, thereby promoting the expression of FTH1 and FTL (46). This study demonstrates that the probability of viral infection is significantly higher in the pSS group than in the HC group, and this process may disrupt cellular iron metabolism, leading to inflammation and cancer. Furthermore, FTH1 participates in the negative regulation of ferroptosis by altering iron metabolism and lipid peroxidation (47). The FTH1/FTL proteins are responsible for helping cells maintain appropriate iron levels, and SCARA5 participates in the positive regulation of cell migration, movement, and growth (48). The interaction between FTH1/FTL and SCARA5 may activate signaling pathways within neutrophils and pDCs, thereby regulating cell proliferation, differentiation, and inflammatory responses (49). Given that pDCs can produce type I IFN via the TLR7/9 signaling pathway (50), interactions between neutrophils and pDCs are hypothesized to regulate the immune functions of both cell types, thereby influencing the inflammatory level and immune status in pSS. When neutrophils serve as receptor cells, compared with the HC group, the chemokine CCL5, which is highly expressed by neutrophils in the pSS group, attracts NK cells expressing the CCR1 receptor. CCL5-CCR1 is a key regulator of immune cell migration and activation in inflammatory autoimmune diseases, capable of inducing the activation and proliferation of specific NK cells while enhancing their cytotoxicity. This interaction plays a crucial role in the immunopathogenesis of pSS (51). Furthermore, compared to the HC group, CCL5-CCR1 upregulation observed in SS salivary glands correlates with increased lymphocyte infiltration and glandular dysfunction in SS, suggesting CCL5-CCR1 may represent a potential therapeutic target (52). In-depth analysis of neutrophil-associated cell communication in pSS disease reveals robust interactions between neutrophils and other cell types. Neutrophils exhibit potent signaling capabilities and function as signal receptors, signaling ligands, bridges, and information conduits to influence other cells.

There is an important methodological limitation in this study that should be noted. This study employed cells separated by Ficoll-Paque density gradient centrifugation for single-cell transcriptomics analysis; however, due to the high density of neutrophils, this method inherently leads to the depletion of most granulocytes, including neutrophils. Nevertheless, neutrophil-like populations were still detected in our single-cell dataset. Several factors may account for this observation. First, under pathological conditions such as inflammation, infection, or autoimmune diseases, neutrophils can undergo activation-induced density changes. Emerging evidence has demonstrated the existence of low-density neutrophils (LDNs) and granulocytic myeloid-derived suppressor cells (G-MDSCs), which co-purify with PBMCs following density gradient centrifugation (53–55). Second, despite the depletion of the majority of neutrophils, a small residual fraction may persist in the PBMC layer under standard isolation conditions, enabling their detection in downstream single-cell analyses. Importantly, we acknowledge that the use of a PBMC isolation protocol for neutrophil-related analyses constitutes a methodological limitation. While the presence of neutrophils in our dataset can be rationalized by the mechanisms described above, this approach does not capture the full heterogeneity of circulating neutrophils, particularly conventional high-density neutrophils. Therefore, interpretations regarding neutrophil biology in the current study should be considered preliminary. Future studies employing optimized neutrophil isolation strategies (such as immunomagnetic selection) will be crucial for validating and extending our findings.

In summary, the findings of this study indicate that various neutrophil subsets participate in different stages of pSS pathogenesis and progression through signaling pathways such as type I IFN. Notably, the up-regulated DEGs *LY6E* and *CLEC12A* in pSS neutrophils may serve as biomarkers and potential therapeutic targets for pSS. This study may offer new insights into the pathophysiological mechanisms of pSS and potential avenues for future therapeutic exploration, though certain limitations exist. Sample size may have influenced the broad applicability and representativeness of the data to some extent. We look forward to more studies in the future to elucidate the role of neutrophils in pSS disease.

## Data Availability

The data presented in the study are deposited in the National Center for Biotechnology Information Sequence Read Archive (SRA) (https://www.ncbi.nlm.nih.gov/sra), identifier: PRJNA1458513.
