# Supplementary material for: Characteristics of peripheral blood neutrophil subsets in patients with primary Sjogren’s syndrome based on single-cell RNA sequencing
Source: Front Immunol. 2026 May 4;17:1820004. doi: 10.3389/fimmu.2026.1820004 (PMC13180747; doi:10.3389/fimmu.2026.1820004)
Supplement: Supplementary file 1 [file SupplementaryFile1.pdf]

Supplementary Fig S1

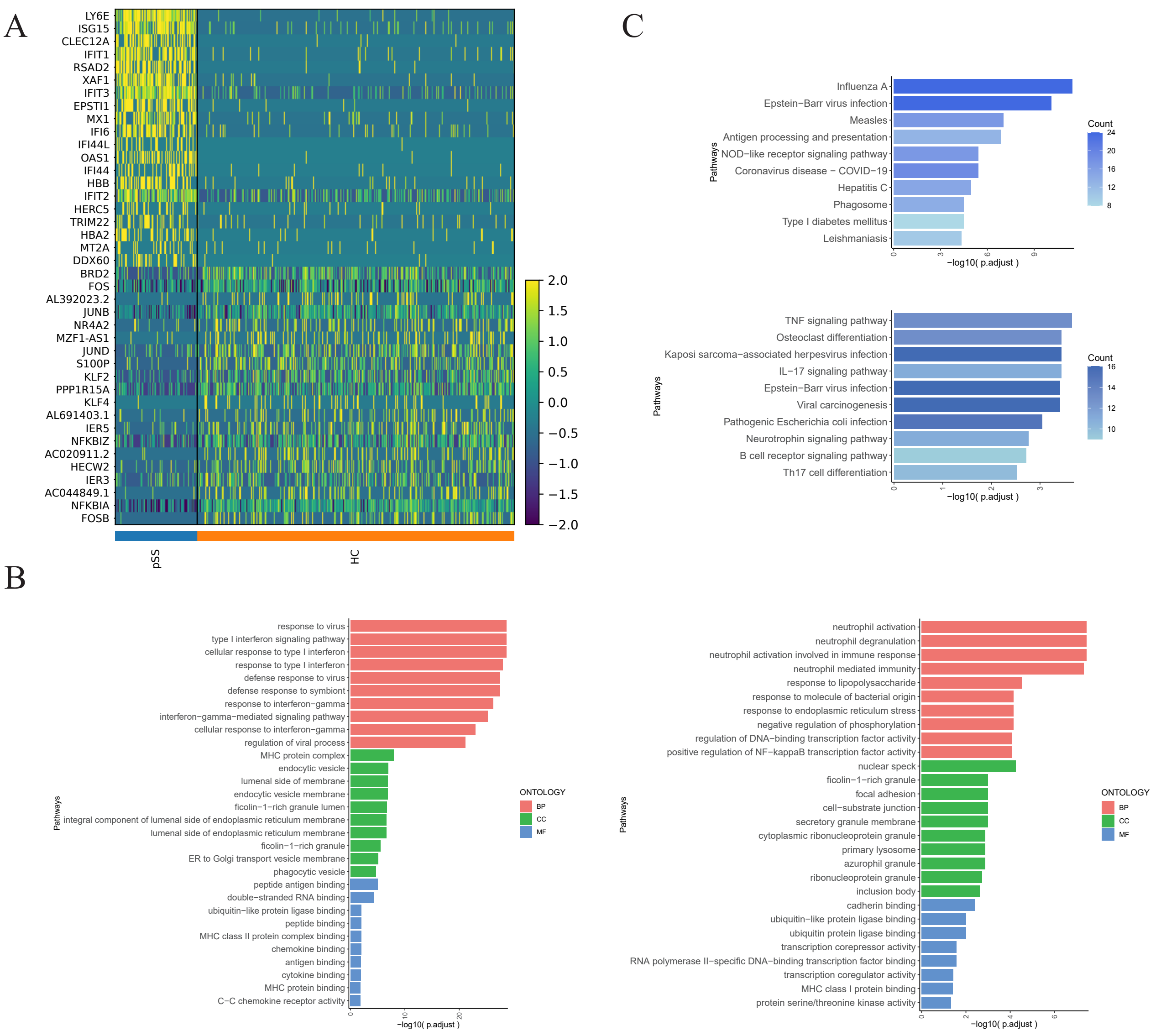

Supplementary Fig S1 | Differential Gene Enrichment Analysis of Neutrophil 1 Subset in the pSS group

(A) Heatmap of the top 20 upregulated and top 20 downregulated genes sorted by score among Neutrophil 1 subset groups between the pSS group and the HC group.

(B) GO enrichment analysis of upregulated genes in the Neutrophils 1 subset of the pSS group (left), GO enrichment analysis of downregulated genes (right). Pathways are displayed on the vertical axis. The longer the column, the more significant the enrichment effect.

(C) KEGG enrichment analysis of upregulated genes of Neutrophils 1 subset in pSS group (top), KEGG enrichment analysis of downregulated genes (bottom).

Supplementary Fig S2

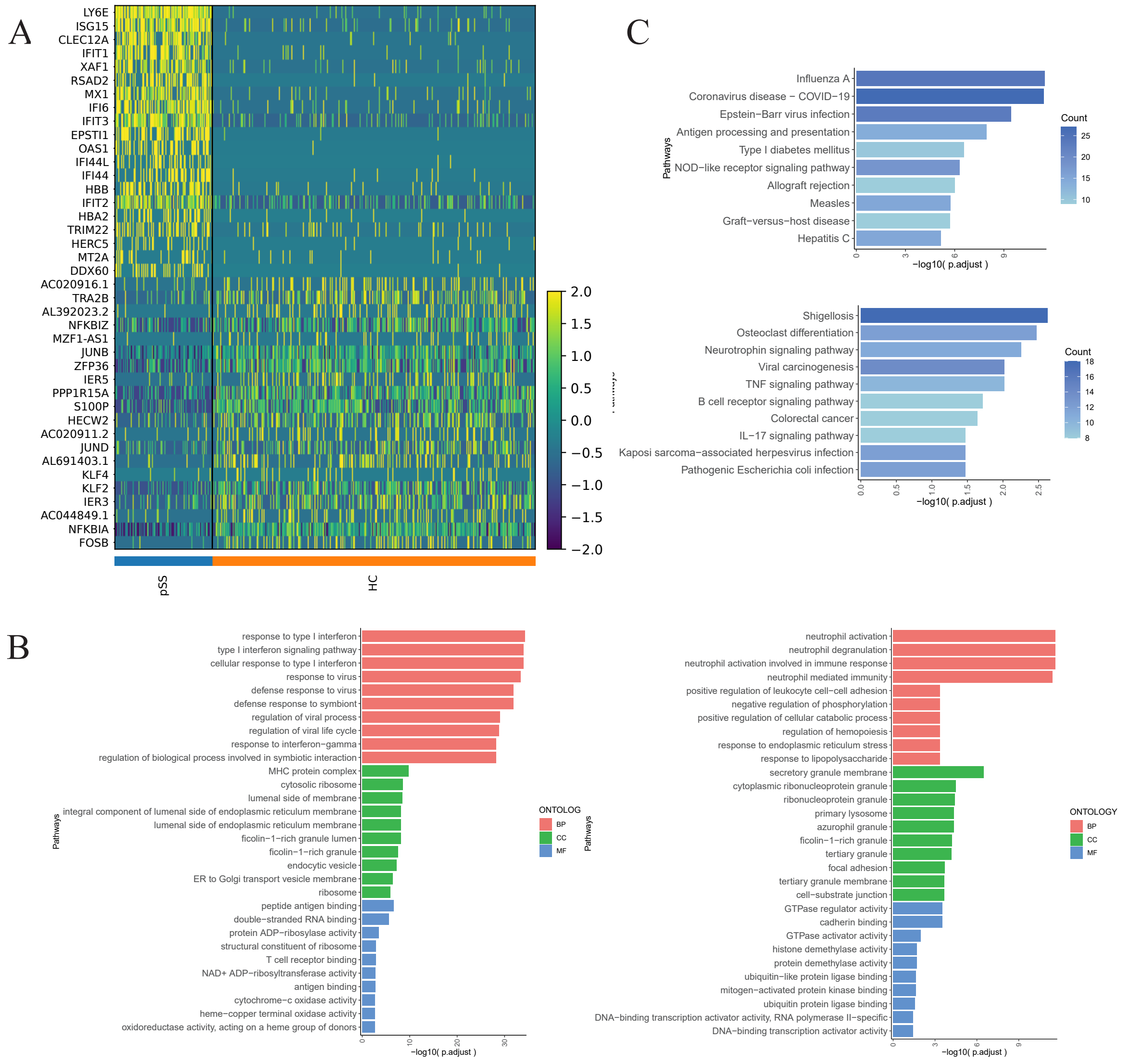

Supplementary Fig S2 | Differential Gene Enrichment Analysis of Neutrophil 3 Subset in the pSS group

(A) Heatmap of the top 20 upregulated and top 20 downregulated genes sorted by score among Neutrophil 3 subset groups between the pSS group and the HC group.

(B) GO enrichment analysis of upregulated genes in the Neutrophils 3 subset of the pSS group (left), GO enrichment analysis of downregulated genes (right). Pathways are displayed on the vertical axis. The longer the column, the more significant the enrichment effect.

(C) KEGG enrichment analysis of upregulated genes of Neutrophils 3 subset in pSS group (top), KEGG enrichment analysis of downregulated genes (bottom).

Supplementary Fig S3

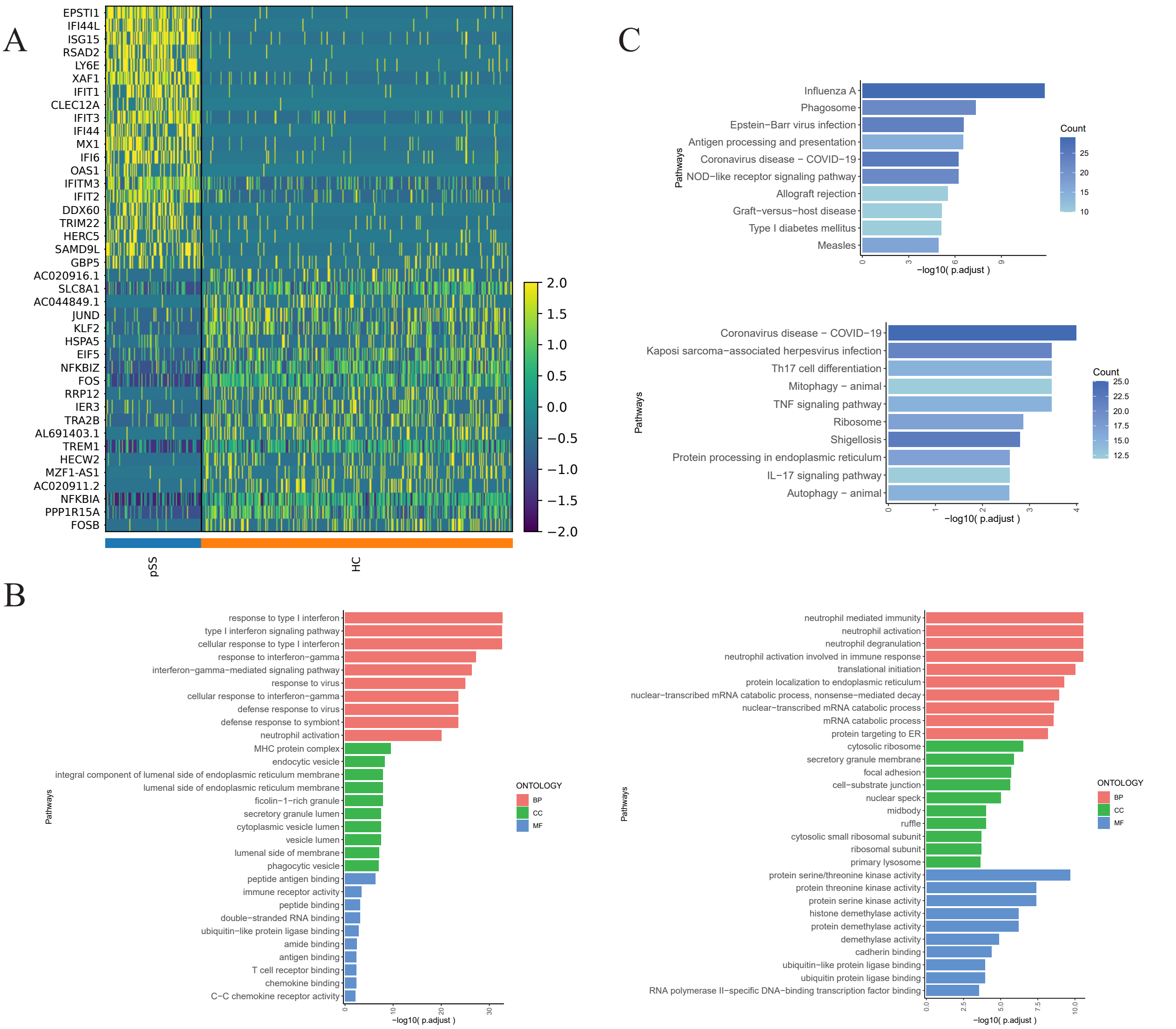

Supplementary Fig S3 | Differential Gene Enrichment Analysis of Neutrophil 4 Subset in the pSS group

(A) Heatmap of the top 20 upregulated and top 20 downregulated genes sorted by score among Neutrophil 4 subset groups between the pSS group and the HC group.

(B) GO enrichment analysis of upregulated genes in the Neutrophils 4 subset of the pSS group (left), GO enrichment analysis of downregulated genes (right). Pathways are displayed on the vertical axis. The longer the column, the more significant the enrichment effect.

(C) KEGG enrichment analysis of upregulated genes of Neutrophils 4 subset in pSS group (top), KEGG enrichment analysis of downregulated genes (bottom).

Supplementary Fig S4

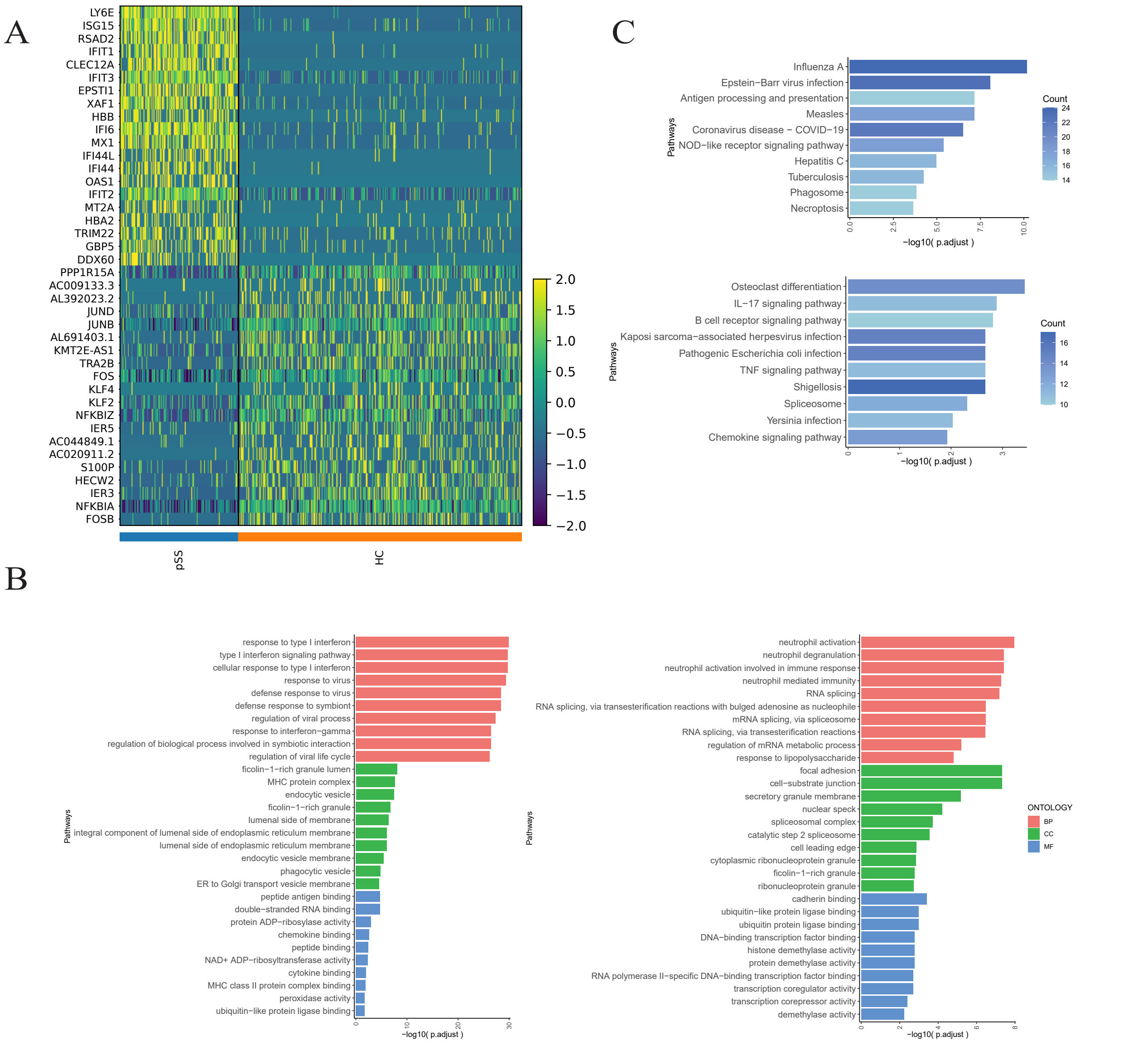

Supplementary Fig S4 | Differential Gene Enrichment Analysis of Neutrophil 5 Subset in the pSS group

(A) Heatmap of the top 20 upregulated and top 20 downregulated genes sorted by score among Neutrophil 5 subset groups between the pSS group and the HC group.

(B) GO enrichment analysis of upregulated genes in the Neutrophils 5 subset of the pSS group (left), GO enrichment analysis of downregulated genes (right). Pathways are displayed on the vertical axis. The longer the column, the more significant the enrichment effect.

(C) KEGG enrichment analysis of upregulated genes of Neutrophils 5 subset in pSS group (top), KEGG enrichment analysis of downregulated genes (bottom).

Supplementary Fig S5

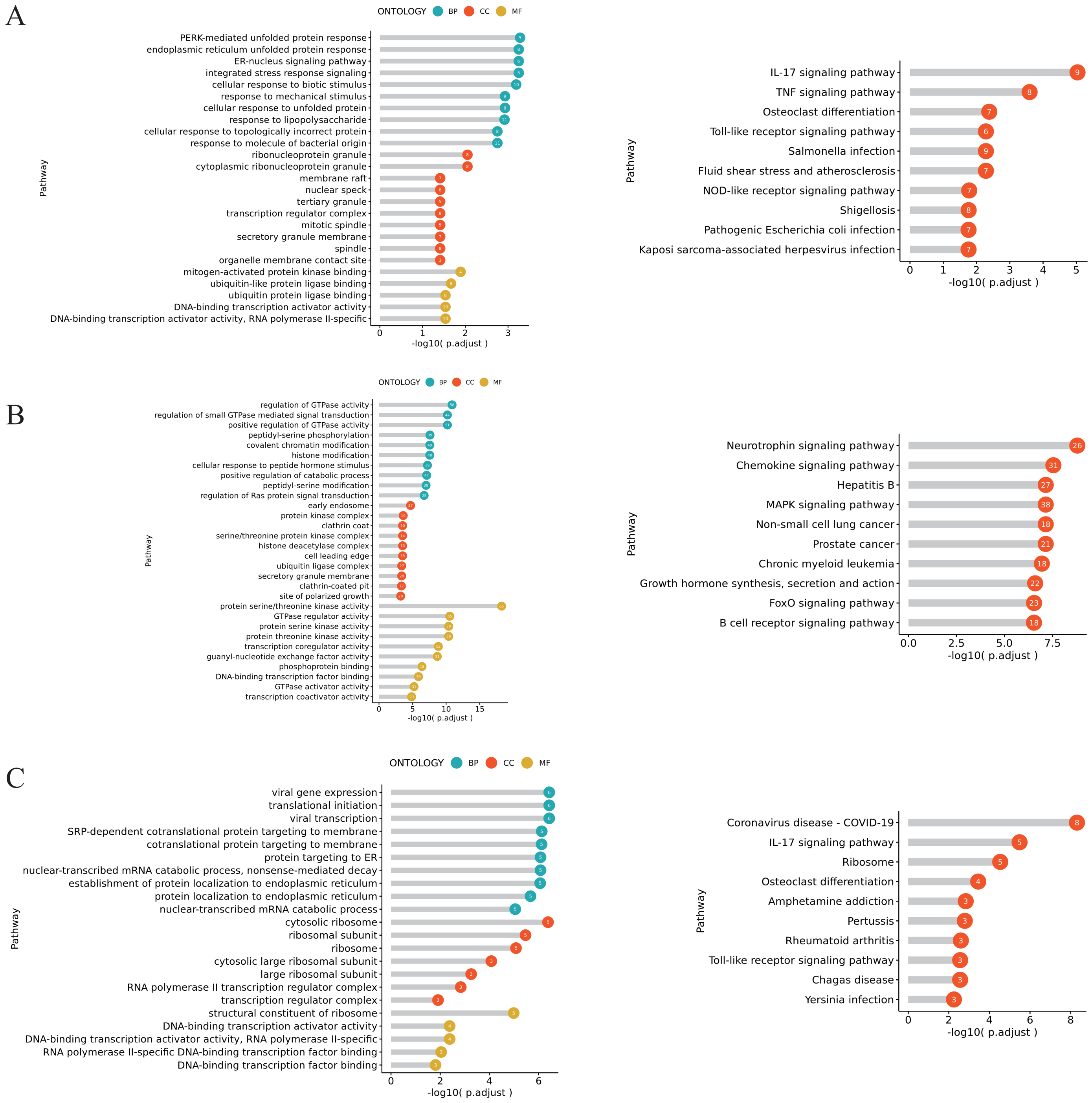

Supplementary Fig S5 | Functional enrichment of DEGs in Neutrophil subsets in the pSS group and the HC group

(A) GO and KEGG enrichment analysis for the Neutrophils 1 subset. The x-axis represents -log10 (p.adjust), while the y-axis shows enriched pathways. Longer lollipop bars indicate more significant enrichment, and numbers within circles denote the number of genes enriched in that pathway.

(B) GO and KEGG enrichment analysis for the Neutrophils 4 subset.

(C) GO and KEGG enrichment analysis for the Neutrophils 5 subset.
